# Effectiveness of Digital Health Interventions on Sedentary Behavior Among Patients With Chronic Diseases: Systematic Review and Meta-Analysis

**DOI:** 10.2196/59943

**Published:** 2025-06-24

**Authors:** Yan Zhang, Fei Wan Ngai, Qingling Yang, Yao Jie Xie

**Affiliations:** 1 Department of Cardiology, Affiliated Hospital of Qingdao University, Qingdao, China; 2 School of Nursing, Faculty of Health and Social Sciences, The Hong Kong Polytechnic University, 11 Yuk Choi Rd, Hung Hom, Hong Kong, 999077, China, 852 34003798, 852 23649663; 3 Research Centre for Chinese Medicine Innovation, The Hong Kong Polytechnic University, Hong Kong, China

**Keywords:** digital health intervention, sedentary behavior, physical activity, chronic diseases, PRISMA

## Abstract

**Background:**

Individuals with chronic diseases commonly engage in a sedentary lifestyle, which may exacerbate poor disease progression and increase the burden of care. Digital health interventions have been broadly used in promoting healthy lifestyles in recent decades, while their effectiveness on sedentary behavior (SB) remains inconsistent and inconclusive.

**Objective:**

This review aimed to evaluate the effectiveness of digital health interventions in reducing SB among patients with chronic diseases.

**Methods:**

PubMed, Embase, Scopus, Web of Science, CINAHL Complete, Cochrane Library, and ACM Digital Library were searched for randomized controlled trials published from January 2000 to October 2023. Two researchers independently screened studies and evaluated study quality. The revised Cochrane risk-of-bias tool was used to assess the risk of bias. Mean differences (MDs) were calculated for intervention effect comparison.

**Results:**

Twenty-six trials were selected and 3800 participants were included. The mean age was 57.32 (SD 9.91) years. The typical chronic diseases reported in the studies included obesity (n=6), arthritis (n=5), coronary artery disease (n=4), cancer (n=4), type 2 diabetes mellitus (n=3), metabolic syndrome (n=2), and stroke (n=2). Phone, web, and activity trackers were 3 digital technologies adopted in the interventions and they were used in combination in most studies (18/26, 69.2%). The functions included facilitating self-monitoring of SB, reminding interruption of long undisturbed sitting, and promoting goal attainment. Approaches targeting SB reduction included standing (n=6), walking (n=9), light physical activity (n=5), moderate to vigorous physical activity (n=4), screen time limitation (n=2), and contextual-related activities based on patients’ preference (n=4). The majority (80.8%) of studies had a low to moderate risk of bias. Meta-analysis revealed significant decreases in overall sitting time (MD −30.80; 95% CI −49.79 to−11.82; *I*^2^=65%; *P*=.001), pre-post sitting time changes (MD −50.28; 95% CI −92.99 to −7.57; *I^2^*=92%; *P*=.02), and SB proportions (MD −4.65%; 95% CI −7.02 to −2.28; *I^2^*=20%; *P*<.001) after digital health interventions, compared with nondigital interventions such as usual care, wait-list, or other active controls, with a small effect size (Cohen *d*=−0.27 to −0.47). No significant differences in the length of sedentary bouts and breaks were found. Subgroup analyses showed that studies with objective SB measurements and those younger than 65 years had significant reductions in sitting time.

**Conclusions:**

Digital health interventions significantly reduced the SB among patients with chronic illness. More research with rigorous design to promote a long-term decrease in sitting time, differentiate primary and compensatory SB reductions, and explore the underlying mechanisms is needed.

## Introduction

Chronic diseases, including cardiovascular diseases, cancers, chronic respiratory diseases, and diabetes, contributed to 71% of all causes of death, imposing an enormous and growing global burden on health care systems and financial expenditure [1]. Individuals diagnosed with chronic diseases required consistent engagement in physical activity (PA) to prevent disease progression and enhance long-term quality of life [2]. However, those patients were always engaged in substantially long sedentary lifestyle because of limited physical function, fatigue, and insufficient exercise endurance.

Sedentary behavior (SB), defined by the Sedentary Behavior Research Network and the American Heart Association, means any waking behaviors characterized by an energy expenditure of ≤1.5 metabolic equivalents (METs) while in a sitting or reclining posture [3,4]. Patients with chronic diseases continue to demonstrate a high level of SB. For instance, a study investigating 131 patients with coronary artery disease (CAD) demonstrated an average sitting time of 10.4 hours per day [5]. Patients with acute coronary syndrome continued to exhibit prolonged sitting of approximately 9 hours per day even after participating in cardiac rehabilitation [6]. Another study indicated that patients with rheumatoid arthritis (RA) spent 71%‐92% of their waking hours in SB [7]. Among 2497 individuals with type 2 diabetes mellitus (T2DM), they allocated an average of 64% waking hours in SB [8]. Patients with stroke also reported 9.22 hours per day sitting time in the first week after discharge in a prospective cohort study [9]. Similarly, a national survey among 741 survivors from cancer found that the patients spent at least 8 hours per day in sitting [10]. Such long-time SB accelerated the negative progression of diseases in those patients [11-13].

The detrimental effects of prolonged SB on chronic disease progression were attributed to various underlying mechanisms, including increased central arterial stiffness [14], impaired vascular function and structure, reduced antegrade blood flow and shear rate, increased insulin resistance, and heightened oxidative stress and apoptosis [15,16]. Those functional deterioration resulted in suboptimal control of cardiometabolic indicators, subsequently increased occurrence of complications, and elevated risk of hospital readmission and mortality [4,17-19]. Therefore, reducing SB, typically in patients with chronic diseases, is a public health priority.

In recent decades, digital health, or the use of digital technologies for health, has gained significant prominence in addressing various health needs [20]. In 2019, the World Health Organization (WHO) announced the Global Strategy on Digital Health 2020‐2025, which emphasized the adoption of digital health in health care [21]. Digital health interventions have great potential for behavioral change without on-site visits, no time constraints, and few travel costs, which facilitate flexible, efficient, and cost-effective interventions [22]. Currently, many researchers have applied digital health interventions for SB reduction. However, the results of previous studies were still inconsistent and inconclusive. For example, a meta-analysis indicated that wearable activity trackers were associated with a significant reduction of 35.46 minutes per day in sedentary time among hospitalized patients, but this finding was derived from only 2 studies included in this review [23]. Additionally, 2 other systematic reviews reported conflicting results regarding the effectiveness of smartphone-based interventions in interrupting SB in older adults, whereas this evidence was also obtained from a limited number of studies (n≤2), and the definition of SB was different [24,25].

Given the rapid development in digital technologies and variation of intervention patterns, as well as the importance of improving SB among patients with chronic diseases, it is crucial to update and comprehensively summarize the evidence to incorporate digital solutions for this vulnerable population. Thereby, this systematic review and meta-analysis aimed to search and synthesize available evidence regarding the effectiveness of digital health interventions in improving SB among patients with chronic diseases.

## Methods

This systematic review and meta-analysis was registered in the PROSPERO (no. CRD42023477958). The PRISMA (Preferred Reporting Items for Systematic Reviews and Meta-Analyses) statement was followed in conducting the review (Checklist 1) [26].

### Inclusion and Exclusion Criteria

The PICOS (Population, Intervention, Control, Outcome, and Study) framework was used to select eligible studies.

#### Population

The population included adults diagnosed with chronic diseases in terms of cardiovascular disease, chronic respiratory disease, cancer, diabetes, metabolic syndrome (MetS), obesity, RA, stroke, and other chronic conditions defined by the WHO [27].

#### Intervention

Digital health intervention is a discrete functionality of digital technology that is applied to achieve health objectives according to WHO guideline [20]. Therefore, interventions delivered using any type of digital technologies, consisting of mobile phones, web, software apps, wearable trackers, emails, or other digital technologies, were included. Studies that exclusively used wearable trackers to measure SB were excluded. The interventions should directly target SB reduction or PA levels increase, which may indirectly result in compensatory changes in SB [28].

#### Control

This included any comparison without digital technologies, including usual care, wait-list, active control, or blank control.

#### Outcome

The definition of SB from the Sedentary Behavior Research Network was adopted [29]. Any SB-related outcomes, including overall sitting time, pre-post sitting time changes, SB proportion, sedentary bouts, and breaks of prolonged sitting, which were assessed at baseline and end point using objective or subjective measures, either as primary or secondary outcomes, were involved.

#### Study

The study types included randomized controlled trials (RCTs). Pilot RCTs, feasibility studies, protocol papers, brief reports of RCTs, and studies with a sample size <10 were excluded.

### Search Methods

The search strategy was developed by 1 author (YZ) and then reviewed and finalized by 2 experts (YJX and FWN) (Multimedia Appendix 1). Two keywords were first defined to develop MeSH (Medical Subject Headings) terms in the search strategy: SB and digital technology. To find patients with any potential chronic illness, no keyword, in particular, for any chronic disease was used in the search strategy. We conducted a systematic search to retrieve all papers focusing on SB interventions using digital health technologies. Two independent authors (YZ and QLY) manually screened these papers and identified those specifically targeting patients with chronic diseases, as defined by the *International Classification of Diseases, Eleventh Revision* (*ICD-11*). At last, a comprehensive search was carried out in PubMed, Embase, Scopus, Web of Science, CINAHL Complete, Cochrane Library, and ACM Digital Library. The papers in English published from 2000 onward were included, which aligned with the first release of the WHO’s document on the approach to digital health strategies [21].

### Study Selection and Data Extraction

Duplicate studies were identified and removed using the NoteExpress software (Beijing Aegean Software). The remaining studies were screened independently by 2 authors (YZ and QLY) in a sequential manner of title, abstract, and full-text screening. Conflicts were settled by consulting a third author (YJX). A standardized data extraction form was used to collect the data on study characteristics (authors, publication years, participants’ demographic details, and dropout rates), intervention details (content, type of digital technology, and duration of the intervention), and outcomes (SB-related outcomes and other primary and secondary outcomes). The data extraction process was conducted independently by 2 authors (YZ and QLY). Any discrepancies in data interpretation were discussed and adjudicated by a third author (YJX).

### Study Quality Assessment

The risk of bias was assessed using the “Revised Cochrane risk-of-bias tool for randomized trials” (RoB2) [30]. Five domains with signaling questions were evaluated, including the randomization process, the effect of assignment and adherence to the intervention, missing outcome data, outcome measurement, and the selection of reported results. Each criterion was assessed and categorized as “low risk,” “some concern,” or “high risk” of bias. An algorithm based on these 5 domains was used to determine the overall bias. Two independent reviewers (YZ and QLY) evaluated the selected papers, and any disagreements were resolved by a third party (YJX).

### Statistical Analysis

The data analysis was conducted using Review Manager 5.3. Twenty-two studies used objective activity trackers to quantify SB-related outcomes, enabling the use of mean differences (MDs) to effectively highlight absolute differences in the mean values of these outcomes [30,31]. Cohen *d* was used to assess the effect size, with *d*>0.8 indicating a large effect, 0.5‐0.8 a medium effect, and 0.2‐0.5 a small effect [32]. For studies with multiple assessments of SB outcomes in 1 study, the final assessment point was selected. Objective SB outcomes (eg, daily sedentary time measured by accelerometer) were prioritized to minimize measurement heterogeneity. The heterogeneity among studies was assessed using the *I*^2^, categorized as low (*I*^2^<25%), medium (25%≤ *I*^2^<75%), or high (*I*^2^≥75%) [33]. Forest plots were used to visually compare the estimated effects and CIs. Funnel plots were used to evaluate publication bias when there were at least 10 studies in the meta-analysis [30]. Egger test was conducted using Stata (version 12; Stata Corp). Sensitivity analyses with leave-one-out principle were performed to assess the robustness of the main results. In cases where quantitative synthesis was not appropriate, a narrative synthesis was conducted.

## Results

### Study Selection

**Figure 1.**
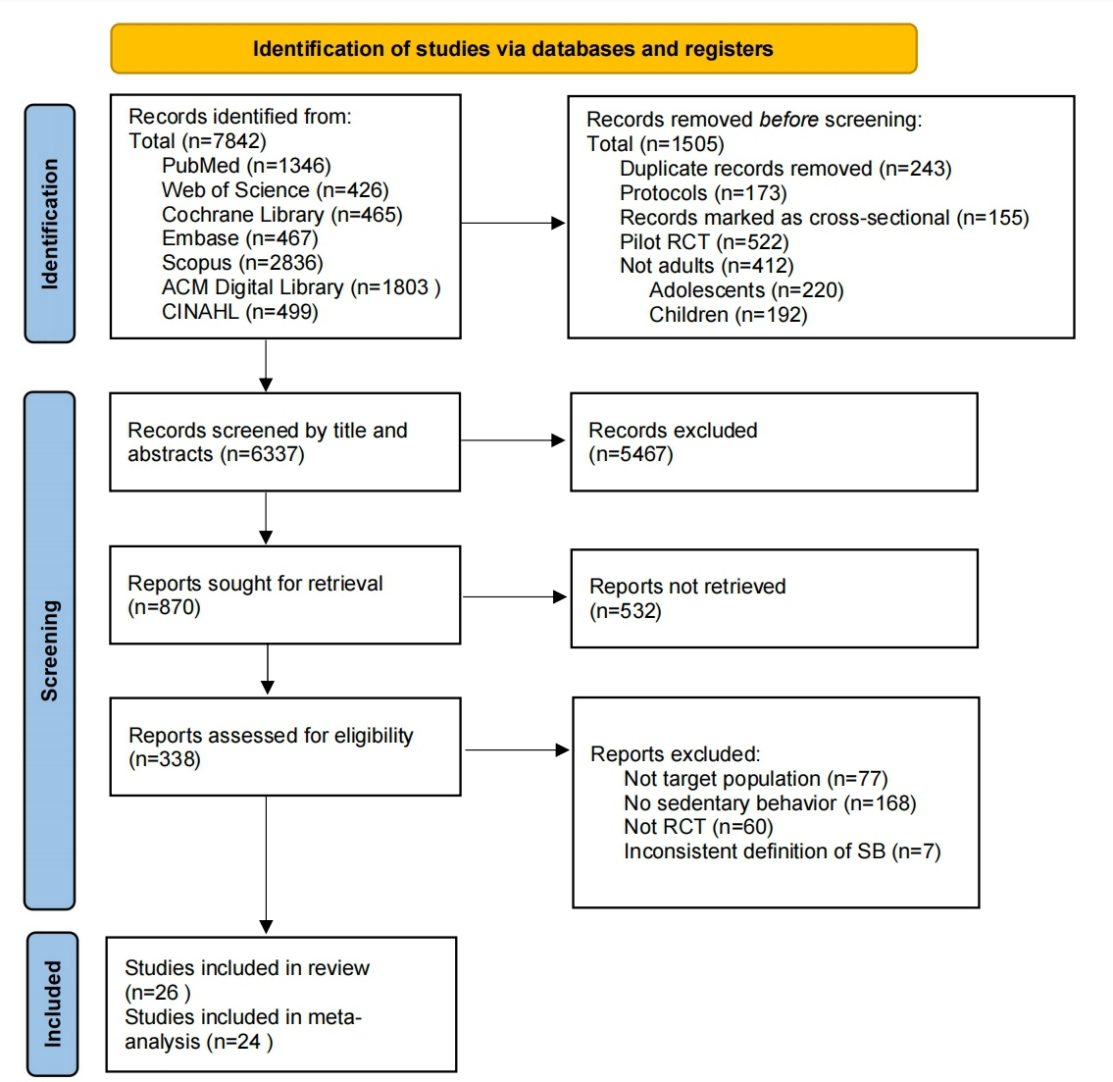
PRISMA (Preferred Reporting Items for Systematic Reviews and Meta-Analyses) flow diagram of the literature search and selection process. RCT: randomized controlled trial; SB: sedentary behavior.

A total of 7842 studies were identified after the systematic search. After removing duplicates, 6337 papers were screened by title and abstract. Ultimately, 338 papers were retrieved and assessed for eligibility through full text review. Of these, 312 papers were excluded due to reasons such as nontarget population, inappropriate study design, or inconsistent definitions of SB, such as TV watching, physical inactivity, or steps. The excluded studies are listed in Multimedia Appendix 1. Figure 1 shows the selection process. Among the included studies, 4 studies were based on 2 separately registered RCTs with different sample sizes or follow-up duration [34-37], and all these studies were then included. While for the other 3 RCTs based on the same study [38-40], only 1 was selected as representative [38], because it closely matched the registered content, also published earlier, and directly focused on SB interventions. In total, 26 studies were identified. Details of the selected papers are summarized in Table S1 in Multimedia Appendix 2.

### Study Characteristics

#### Study Settings, Design, and Population Characteristics

All the included studies were published in English between 2011 and 2023, with the majority in Western countries (16/26, 61.5%). The Netherlands [41-44] and Australia [45-48] had the same and highest number of studies (n=4). There were 2 three-arm RCTs [49,50] and 1 four-arm RCT [28], and all others were 2-arm RCTs. The sample size varied from 51 to 622, with a total of 3800 participants involved. The average age of the participants was 57.32 years (SD 9.91). There were more women (n=2042) than men (n=1758), while 2 studies specifically focused on women [46,51]. The chronic diseases included obesity (n=6) [28,38,41,42,50,52], RA (n=5) [36,37,44,53,54], CAD (n=4) [43,48,55,56], cancer (n=4) [45-47,51], T2DM (n=3) [49,57,58], MetS (n=2) [59,60], and stroke (n=2) [34,35]. All the included studies used digital-assisted interventions using various technologies at different levels. Most control groups were assigned to usual care (n=13), maintained their usual lifestyle (n=5), or were placed on a wait-list control (n=4).

#### SB-Related Outcomes

Sixteen studies designated SB as the primary outcome [28,34-37,42,43,45-47,49,51,52,58-60], while the remaining 10 RCTs identified PA as the primary outcome [44,53-57,60-63]. SB-related outcomes included the overall sitting time (n=16), pre-post sitting time changes (n=8), SB proportion among all activities (n=4), sedentary bouts (n=7), and breaks of sedentary time (n=6). The overall sitting time (minutes per day) was the volume of sitting time accrued per day, which was the most common indicator in SB assessment [64]. The pre-post sitting time changes (minutes per day) were calculated as the overall sitting time after intervention minus the baseline sitting time, also called sedentary time reduction [43]. SB proportion (%) was the percentage of time spent performing the activities that require ≤1.5 MET of tasks, after the subtraction of sleeping time [34,35]. Essentially, it also referred to the SB percentage among all activities, including SB (MET ≤1.5), light physical activity (LPA) (1.5 < MET ≤3), and moderate to vigorous physical activity (MVPA) (MET >3.0) [28].

Sedentary bout was defined as a continuous period of uninterrupted sedentary time. However, there seemed to be a lack of consensus on the criteria used to determine the sedentary bouts. Six studies used various thresholds for defining sedentary bouts, including 30 minutes [43], 20 minutes [46,47,53,54], and 10 minutes [49]. And 1 study reported the average duration of all sedentary bouts [48]. Six studies presented data on breaks of sedentary time (frequency of interrupted sedentary time per day), using the number of breaks [38,48,49,52], and changes in the number of breaks from baseline to end point [36,37]. However, only 1 study reported how to determine the “break” clearly, stating that a break was defined as a transition from SB (<99 counts per minute, measured by accelerometer; ActiGraph) to any other level of PA (>99 counts per minute) between 2 sedentary bouts (>10 consecutive minutes) [49].

In terms of measurement of SB, most of the researchers (22/26, 84.6%) used objective activity trackers. Four studies adopted subjective methods, including the International Physical Activity Questionnaire [56,58], the Activity Questionnaire for Adolescents and Adults [42], and telephone interviews [45]. Only 2 studies used both an accelerometer and a questionnaire [51,58].

#### Characteristics of Digital Health Interventions

The intervention primarily targeting SB encompassed screen time limitation (n=2) [34,35], standing (n=6) [36,38,43,46,52,59], walking (n=9) [36,43,45-47,49,51,52,60], LPA (n=5) [37,38,45,51,59], and MVPA (n=5) [28,38,42,47,60]. The majority of these trials (11/16, 68.8%) adopted the combination of these approaches. Regarding SB intervention, the context in which the SB occurred included leisure time, household tasks, transportation, and occupational settings [65]. Due to this context-related characteristic of SB, 4 RCTs explicitly proposed that participants could select the methods and set goals of interrupting SB based on their preference, including using standing desks, standing during phone calls, taking stairs instead of elevators, home-cleaning activities, or other kinds of LPA [36,38,43,59]. Six studies reported the compensatory SB reduction by increasing PA level (without addressing SB directly) [28,44,50,55-57], which included LPA/MVPA [50,55,57], cardiac rehabilitation [56], and free choice of any kinds of exercise, such as gardening, cycling [44], endurance, and strength training [28]. Motivational counseling or health coaching was also frequently used (8/16, 50%) to assist participants in increasing awareness of the detrimental effects of SB, establishing and revising goals, or receiving personalized prescriptions based on their preferences [36,37,45,46,51,52,58,59].

More than one-third of the studies (10/26, 38.5%) adopted a theoretic framework in intervention design and implementation. Three of them applied the Social Cognitive Theory [36,56,60], incorporating pivotal constructs such as self-efficacy and outcome expectations. This theory emphasized optimization of cognitive, behavioral aspects (eg, self-efficacy and skill mastery), and environmental influences (eg, peer support and barriers to action) to facilitate behavioral modifications. Two studies applied the Self-regulation Theory, which reinforced an individual’s attitude and behavioral intention to change. Moreover, the discrepancy between a person’s goal and the actual situation also served as a catalyst for behavioral transformations [42,49]. Other studies were based on the principle of Self-efficacy Theory [37], Theory of Planned Behavior [42], Acceptance Commitment Therapy [45], intervention mapping adaption framework [43], or Cognitive Behavioral Therapy [58]. In summary, these theories were used to facilitate the reduction of SB or enhancement of PA by influencing individuals’ attitudes, beliefs, motivations, and cognitive processes. The intervention duration ranged from a minimum of 6 weeks to a maximum of 24 months, with a median duration of 6 months. Three months accounted for the highest proportion (7/26, 26.9%).

#### Digital Technologies Used in the Interventions

Three kinds of digital technologies were adopted in the intervention, including phone (phone calls, text messages, and apps), web (email and web-based messages), and activity trackers (accelerometer, pedometer, and heart rate monitor). Two studies used all 3 types of digital technologies [53,54], while 16 RCTs used 2 kinds [28,38,41,43,44,46-52,56,57,59,60] and 8 studies used 1 type such as phone calls (n=5), text messages (n=2), and web (n=1) [34-37,42,45,55,58]. Phone-based digital health interventions were the most prevalent (22/26, 84.6%), including phone calls and short text messages, which were applied to remind participants to adhere to the interventions. Two studies used common phone-based apps, such as WhatsApp [57] and WeChat [56], for liaison with participants, while others developed tailored ones, such as Mijn Actieplan [49] and Heathesteps [60], to support self-monitoring, reminding, and goal attaining. One study stated that they used a virtual coach in the smartphone app to provide personalized prescriptions and help participants maintain their goals [60]. However, details were limited even in the protocol [66].

Web-based interventions accounted for 26.92% (7/26) in all studies, and more than half of the interventions (4/7, 57.10%) were in combination with phones to facilitate the goal attainment process or disseminate supportive information [49,54,56,60]. It also served as a management platform for researchers to access participants’ SB status in a real-time manner, which enabled timely feedback, information sharing, and encouragement to attain the designed goals [55].

Half of the studies (13/26, 50%) used activity trackers, including wearable accelerometer (wrist, hip, or pocket-worn) [38,41,43,46-48,52-54], pedometer [51], and heart rate monitor [51], for data collection, self-monitoring, and safety assurance. Two of them implemented alerts in the accelerometer using vibrotactile feedback [43] or visual “move” display with an audible beep [46], which directly and efficiently reminded patients about their unconscious long-time sitting. Six studies synchronized activity trackers with phone-based apps via Bluetooth or GPS automatically, enabling researchers or health professionals to provide timely feedback or help participants to self-monitor their behavior, such as SB or PA [38,43,46,48,53,59].

### Risk of Bias

The 80.8% (21/26) of the studies had a low to moderate risk of bias [28,35-38,41,43-47,50,51,53-60]. Domains assessed as having a high risk of bias were attributed to the missing outcome data. Three papers had no description of the allocation concealment [28,50,58]. One study had unbalanced baseline data regarding marital status and employment status (*P*<.05) [57]. One study reported longer sitting time in the intervention group than in the control group (9.8 vs 8.8 hours per day) at baseline [37]. No study adopted blinding because it was not possible due to the nature of the intervention. The majority of the studies used the intention-to-treat analysis, while 3 studies applied the on-protocol analysis [28,50,59]. For the domain of missing outcome data, only 8 of the studies reported a data availability of ≥95% [37,38,43,46,47,53,54,58]. Most of the studies were in line with existing published protocols, except for 3 papers. Two of them decreased sample size owing to the impact of COVID-19 [34,48], and 1 modified the recruitment strategy as there were not enough individuals from the target population [49]. Details regarding the risk of bias for each study and the summary could be found in Multimedia Appendix 3.

### Meta-Analysis

Among the 26 included studies, 24 RCTs were included in the meta-analysis. Rest of the 2 studies just provided limited information and were therefore used only for narrative analysis [59,60].

#### Effects of Digital Health Interventions on Reducing Overall Sitting Time

**Figure 2.**
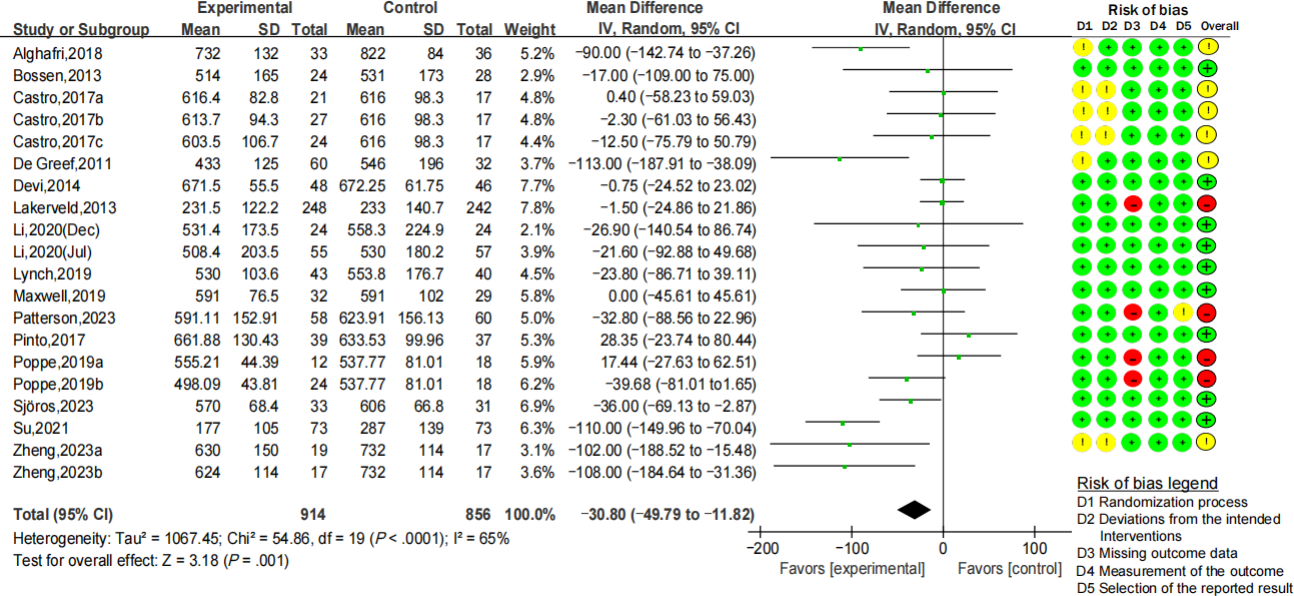
Forest plot for pooled mean difference of overall sitting time (minutes per day) [28,38,42,44,46-51,53-58].

Sixteen RCTs reported the overall sitting time (minutes per day), including 914 participants in the intervention group and 856 in the control group. The overall sitting time was significantly decreased with a pooled MD of 30.80 minutes per day (95% CI −49.79 to −11.82; *I*^2^=65%; *P*=.001) after the interventions (Figure 2), with a small effect size (Cohen *d*=−0.27; 95% CI −0.44 to −0.11; *Z*=3.25; *P*=.001). The study weight, MD of overall sitting time, and mean age of the participants were shown in a grouped bubble plot (Multimedia Appendix 4).

Subgroup analyses were conducted based on disease classification, age group, theoretical framework, primary or compensatory SB reduction, intervention duration, and objective or subjective measurement of SB. There was no significant reduction in overall sitting time (minutes per day) among patients with specific kinds of chronic diseases, including obesity, RA, CAD, cancer, T2DM, MetS, and stroke. As for age groups, only those aged 65 years and younger showed a noticeable decline in sitting time (MD −35.74 minutes per day; 95% CI −57.20 to −14.28; *I*^2^=66%; *P*=.001). Both theory-based and non–theory-based interventions had a significant effect on reducing sitting time, while the theory-based interventions demonstrated a greater reduction (MD −52.48 vs −17.20 minutes per day). A meta-analysis was conducted separately for studies primarily focusing on SB or PA (compensatory SB reduction). The results showed that compensatory SB exhibited a significant decrease (MD −47.58 minutes per day; 95% CI −84.94 to −10.22; *I*^2^=77%; *P*=.01). Regarding the duration of interventions, both the short duration (≤3 months) and the long duration (>3 months) significantly decreased sitting time (MD −55.89 minutes per day; MD −18.86 minutes per day). One study reported an average sedentary time of 18.8 hours based on objective measurement, which was clinically unreasonable and might include sleeping time [58]. Therefore, the subjective data on sedentary time was extracted in this study. The results showed that only those objectively measured sedentary time reduced 22.20 minutes per day significantly (95% CI −39.07 to −5.33; *I*^2^=42%; *P*=.01). Details of the subgroup analyses are shown in Multimedia Appendix 5.

#### Pre-Post Sitting Time Changes After Digital Health Interventions

**Figure 3.**
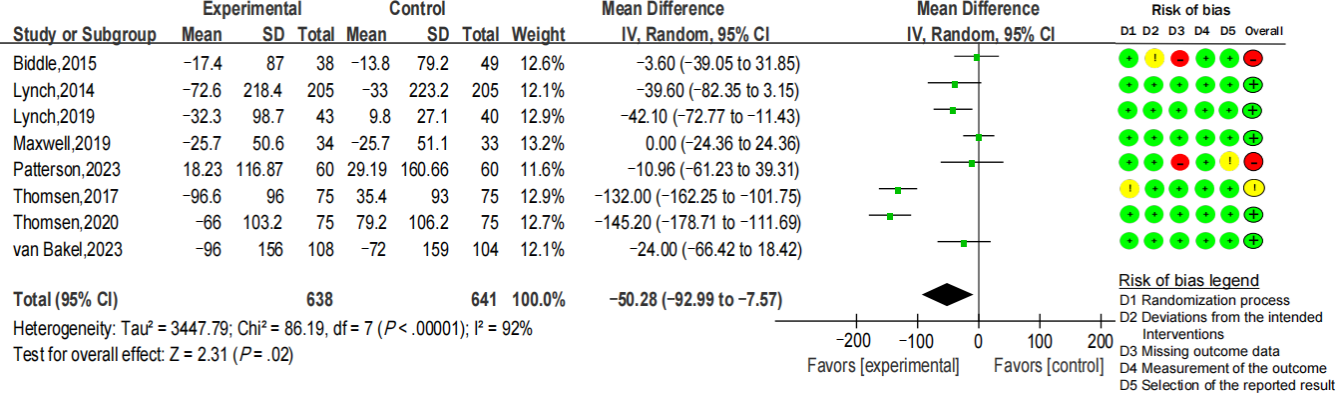
Forest plot for the mean difference of sitting time changes (minutes per day) after intervention [36,37,43,45-48,52].

As depicted in Figure 3, 8 RCTs reported pre-post sitting time changes, involving 638 participants in the intervention group and 641 participants in the control group. The results showed significant changes in sitting time, with an average decrease of 50.28 minutes per day (95% CI −92.99 to −7.57; *I*^2^=92%; *P*=.02), and a small effect size (Cohen *d*=−0.47, 95% CI −0.86 to −0.09; *Z*=2.40; *P*=.02).

#### SB Proportion of All Activities After Interventions

**Figure 4.**
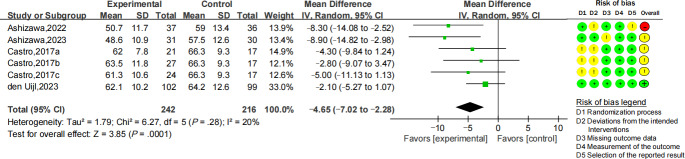
Forest plot for the mean difference of sedentary behavior proportion among all activities (%) [28,34,35,41].

Four RCTs reported SB proportion results. The forest plot showed a pooled MD of 4.65% reduction in favor of the digital health interventions (95% CI −7.02 to −2.28; *I*^2^=20%; *P*=.0001) (Figure 4), with a small effect size (Cohen *d*=−0.40; 95% CI −0.61 to −0.20; *Z*=3.91; *P*<.001).

#### Effects of Digital Health Interventions on Sedentary Bouts and Breaks of Sedentary Time

**Figure 5.**
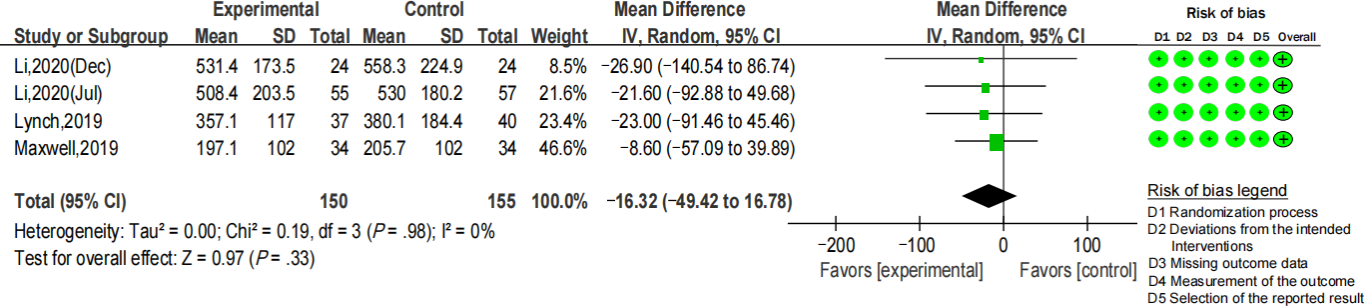
Forest plot for the mean difference of sedentary bouts (>20 minutes per day) [46,47,53,54].

**Figure 6.**
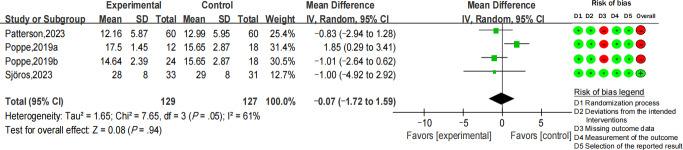
Forest plot for the mean difference of breaks of sedentary time (numbers per day) [38,48,49].

There was no significant decline observed in sedentary bouts >20 minutes (MD −16.32 minutes per day; 95% CI −49.42 to 16.78; *I*^2^=0; *P*=.33) (Figure 5). Among 4 studies that reported the breaks of sedentary time [38,48,49,52], 1 of them presented unreasonable data regarding the breaks without explanation [52]. Therefore, only 3 RCTs were included in the meta-analysis. There was also no significant difference between the intervention and control groups (MD −0.07; 95% CI −1.72 to 1.59; *I*^2^=61%; *P*=.94) (Figure 6).

#### Publication Bias and Sensitivity Analysis

**Figure 7.**
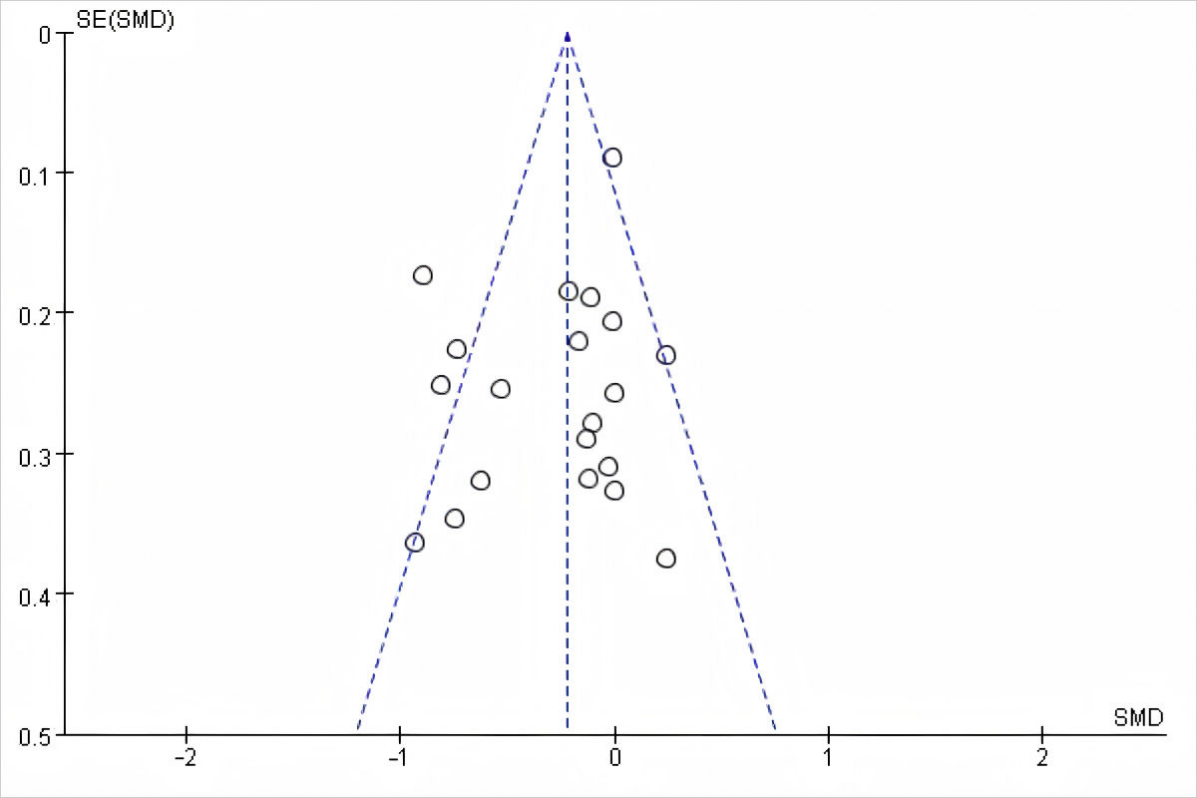
Funnel plot overall sitting time (minutes per day), SMD: standard mean difference.

There was no serious publication bias indicated by the symmetry of the funnel plot for overall sitting time (Figure 7). Egger’s estimated bias coefficient was −1.22 (*P*=.24) and supported that there was no significant evidence of publication bias. Leave-one-out sensitivity analysis showed consistent results in terms of overall sitting time, pre-post sitting time changes, SB proportion, sedentary bouts, and breaks of sedentary time, implying the robustness of the key results.

## Discussion

### Principal Findings

The findings of this review supported the effectiveness of digital health interventions in modifying SB, leading to a reduction in overall sitting time, pre-post sitting time changes, and SB proportion among patients with chronic diseases. The use of digital technology, including mobile phone, web, and activity trackers, facilitated real-time self-monitoring, personalized feedback, and automatic reminders, and elevated motivation and engagement in reducing SB.

Previous researches have shown that SB, characterized by low energy expenditure, was associated with an elevated risk of all-cause mortality in a clear dose-response pattern [12]. This correlation was particularly prominent among individuals who led sedentary lifestyles, notably in those with chronic diseases [67]. The advancement of digital technology could improve the efficacy of the intervention implementation [68]. In this review, the overall sitting time decreased by 30.80 minutes per day, which was less than the 42.28 minutes per day reduction in SB observed in a previous meta-analysis evaluating digital technology–enhanced interventions on SB among healthy adults in a workplace setting [69]. This difference may be explained by different populations (healthy vs unhealthy populations) and settings. Gardner et al [70] supported this explanation by noting that workplace SB was more receptive to routinization than nonworkplace SB. Another systematic review and meta-analysis evaluated the effectiveness of interventions using wearable activity trackers to enhance PA and reduce SB in hospitalized patients, indicating an average reduction of 35.46 minutes per day in sitting time [23]. However, a different meta-analysis including 48 papers revealed that wearable activity trackers effectively improved conscious exercise behavior, such as steps and MVPA in daily life, but they were not effective in modifying habitual behaviors, such as SB [71]. Variations in the target population and the diverse array of digital technologies used may contribute to the discrepancies in results across the referenced studies and this study.

There were no confirmed results regarding the effectiveness of digital health interventions on SB among specific chronic disease due to the limited number of available studies (n≤4). This notion was also highlighted in a scoping review that evaluated the efficacy of mHealth interventions in addressing PA and SB among survivors from cancer. The evidence on SB was inconclusive, primarily attributed to the limited number of available studies [72]. In another systematic review and meta-analysis examining mHealth app interventions in reducing sedentary time among older adults, the findings did not achieve statistical significance, possibly due to the limited sample size [25]. Therefore, more studies targeting different kinds of chronic diseases are warranted. The results were more promising for those aged 65 years and younger, which was reasonable that younger patients may be more adaptable and easier to incorporate new technologies into interventions. However, individuals aged older than 65 years were more likely to experience multiple chronic diseases and complex health conditions, making the maintenance of an active lifestyle particularly crucial for them. Hence, future study needs to explore effective strategies to enhance the efficacy of digital health interventions aimed at reducing SB among older adults, such as improving their digital health literacy [73].

Theory-based approaches showed a higher reduction in overall sitting time than no-theory–based interventions. Social Cognitive Theory was the most extensively applied framework. This theory focused on individuals’ perceptions, beliefs, and expectations regarding SB [74], aiming to raise awareness of the harmful consequences of SB, emphasize the benefits of any level of activity, and promote changes in SB [75]. It was also reasonable to observe a greater SB reduction in the short term than in the long term (55.89 minutes per day vs 18.86 minutes per day). This suggested that maintaining SB reduction was still challenging due to the diminishing novelty of technology-mediated interventions [69] and additional efforts were needed to stimulate long-term SB reduction. For instance, individualized cognitive-behavioral telephone supports were proven effective in sustaining the reduction of SB for at least half a year [58]. The involvement of participants’ respective centers in digital health interventions may escalate trust, facilitate information sharing, and encourage seeking support and feedback as necessary for sustained engagement over an extended duration [57]. In addition, improving awareness of SB-related health issues, addressing long sitting time patterns [58], and replacing SB with LPA together with the individual in daily life perspective [36] may prolong the long-term impact of reducing SB. Only the objective sitting time reduction was significant, while the subjective measurements varied a lot and had high heterogeneity (*I*^2^=96%).

SB and PA were 2 of the 3 main categories of 24-hour movement and nonmovement behaviors and were interchangeable with each other [29]. For approaches targeting SB reduction, standing, walking, LPA, and MVPA were the most common substitutes. For studies focused on PA increase solely, we also found a compensatory SB reduction significantly (47.58 minutes per day). However, previous research mentioned that individuals may engage in increased SB after exercise training, which could be attributed to the perception that they earned the right to be sedentary due to their earlier PA [76]. The difference may be explained by different target populations, and further studies are necessary to conclude the difference and interactive mechanism between primary and compensatory SB reduction.

Sedentary bout, an important aspect of SB, showed no significant alteration after digital health interventions. Among the original trials that emphasized the reduction of sedentary bouts or set an upper limit time on sedentary time [47,53,54], only 1 study demonstrated a significant decrease in sedentary bouts (MD −42 minutes per day; 95% CI −83 to −2; *P*=.04) [46]. Notably, sedentary bouts ≥30 minutes were linked to a higher risk of all-cause mortality compared with shorter bouts [77-79]. Furthermore, among individuals with similar total sitting time, frequent interruptions of sitting were associated with lower cardiovascular risks [80]. Given the insufficient focus on breaks in SB (n=3) in the included studies, further research in this area is also warranted.

### Strengths and Limitations

The comprehensive search strategy was solely based on RCTs and most of the selected trials had a low risk of bias. We prioritized the use of objective measures of SB in the meta-analysis, making the results of the meta-analysis more reliable. Moreover, we performed subgroup analyses based on whether the interventions targeted SB directly or indirectly to distinguish primary SB reduction from compensatory changes of SB after PA interventions.

There were also certain limitations. First, this review selected only those papers published in English, which may exist a language bias. Second, the limited number of RCTs included in subgroup analyses posed a challenge in identifying potential sources of bias, particularly when focusing on specific types of chronic diseases. Third, the substantial heterogeneity observed in the contents of the interventions and the joint application of digital technologies constrained the ability to interpret the individual contribution of each approach or technology. Therefore, caution is warranted when interpreting the results, and further research that compares different delivery methods of digital health interventions is necessary to provide more robust evidence. While it is not feasible to conduct a meta-analysis to compare different technologies, the findings emphasized the potential effectiveness of different combinations of digital technologies in reducing SB. In addition, a few publications were identified as study protocols or pilot studies, necessitating their exclusion with the intent to update at a later time.

### Clinical Implications and Future Research

In clinical practice, nurses could leverage phone, web, or activity tracker–based interventions to offer support to sedentary patients with chronic diseases, which may effectively reduce their sitting time and then facilitate better disease management.

The digital health interventions with rigorous design targeting SB reduction remain limited, particularly for certain type of chronic illnesses. Additionally, it is recommended to place a greater emphasis on addressing sedentary bouts and breaks, rather than solely focusing on reducing overall sitting time. Meanwhile, this review reveals a reduction in compensatory SB reduction following PA interventions, which has not been widely studied before. Further research is needed to understand the underlying mechanisms and their implications for behavior modification.

### Conclusions

Digital health interventions based on phone, web, and activity trackers were promising in reducing sitting time among patients with chronic diseases. Screen time limitation, standing, walking, LPA, and MVPA may be used to substitute SB. Future research focuses on sedentary bouts and breaks with rigorous design are necessary, particularly among patients with specific chronic diseases.

## Supplementary material

10.2196/59943 Multimedia Appendix 1 Search strategy and excluded studies.

10.2196/59943 Multimedia Appendix 2 Characteristics of the included randomized controlled trials.

10.2196/59943 Multimedia Appendix 3 Risk of bias in individual studies and summary of risk bias.

10.2196/59943 Multimedia Appendix 4 Grouped bubble plot of the 16 studies.

10.2196/59943 Multimedia Appendix 5 Results of the subgroup analyses.

10.2196/59943 Checklist 1 PRISMA (Preferred Reporting Items for Systematic Reviews and Meta-Analyses) checklist.

## Abbreviations

CAD: coronary artery disease
LPA: light physical activity
MD: mean difference
MeSH: Medical Subject Headings
MET: metabolic equivalent
MetS: metabolic syndrome
MVPA: moderate to vigorous physical activity
PA: physical activity
PICOS: Population, Intervention, Control, Outcome, and Study
PRISMA: Preferred Reporting Items for Systematic Reviews and Meta-Analyses
RA: rheumatoid arthritis
RCT: randomized controlled trial
SB: sedentary behavior
T2DM: type 2 diabetes mellitus
WHO: World Health Organization

## References

[1] Bennett JE, Stevens GA, Mathers CD (2018). NCD Countdown 2030: worldwide trends in non-communicable disease mortality and progress towards Sustainable Development Goal target 3.4. The Lancet.

[2] Allegrante JP, Wells MT, Peterson JC (2019). Interventions to support behavioral self-management of chronic diseases. Annu Rev Public Health.

[3] Sedentary Behaviour Research Network (2012). Letter to the editor: standardized use of the terms “sedentary” and “sedentary behaviours”. Appl Physiol Nutr Metab.

[4] Young DR, Hivert MF, Alhassan S (2016). Sedentary behavior and cardiovascular morbidity and mortality: a science advisory from the American Heart Association. Circulation.

[5] Bakker EA, van Bakel BMA, Aengevaeren WRM (2021). Sedentary behaviour in cardiovascular disease patients: risk group identification and the impact of cardiac rehabilitation. Int J Cardiol.

[6] Ter Hoeve N, Sunamura M, van Geffen ME (2017). Changes in physical activity and sedentary behavior during cardiac rehabilitation. Arch Phys Med Rehabil.

[7] Prioreschi A, Hodkinson B, Avidon I, Tikly M, McVeigh JA (2013). The clinical utility of accelerometry in patients with rheumatoid arthritis. Rheumatology (Oxford).

[8] van der Berg JD, Stehouwer CDA, Bosma H (2016). Associations of total amount and patterns of sedentary behaviour with type 2 diabetes and the metabolic syndrome: the Maastricht Study. Diabetologia.

[9] Wondergem R, Pisters MF, Heijmans MW (2020). Movement behavior remains stable in stroke survivors within the first two months after returning home. PLoS One.

[10] Kim RB, Phillips A, Herrick K (2013). Physical activity and sedentary behavior of cancer survivors and non-cancer individuals: results from a national survey. PLoS One.

[11] Dempsey PC, Matthews CE, Dashti SG (2020). Sedentary behavior and chronic disease: mechanisms and future directions. J Phys Act Health.

[12] Patterson R, McNamara E, Tainio M (2018). Sedentary behaviour and risk of all-cause, cardiovascular and cancer mortality, and incident type 2 diabetes: a systematic review and dose response meta-analysis. Eur J Epidemiol.

[13] Wu J, Fu Y, Chen D (2023). Sedentary behavior patterns and the risk of non-communicable diseases and all-cause mortality: a systematic review and meta-analysis. Int J Nurs Stud.

[14] Huynh QL, Blizzard CL, Sharman JE, Magnussen CG, Dwyer T, Venn AJ (2014). The cross-sectional association of sitting time with carotid artery stiffness in young adults. BMJ Open.

[15] Carter S, Hartman Y, Holder S, Thijssen DH, Hopkins ND (2017). Sedentary behavior and cardiovascular disease risk: mediating mechanisms. Exerc Sport Sci Rev.

[16] Pinto AJ, Bergouignan A, Dempsey PC (2023). Physiology of sedentary behavior. Physiol Rev.

[17] Chau JY, Grunseit AC, Chey T (2013). Daily sitting time and all-cause mortality: a meta-analysis. PLoS One.

[18] Biswas A, Oh PI, Faulkner GE (2015). Sedentary time and its association with risk for disease incidence, mortality, and hospitalization in adults: a systematic review and meta-analysis. Ann Intern Med.

[19] Wilmot EG, Edwardson CL, Achana FA (2012). Sedentary time in adults and the association with diabetes, cardiovascular disease and death: systematic review and meta-analysis. Diabetologia.

[20] (2019). World Health Organization. Recommendations on digital interventions for health system strengthening.

[21] (2021). Global strategy on digital health 2020-2025. World Health Organization.

[22] Blasiak A, Sapanel Y, Leitman D (2022). Omnichannel communication to boost patient engagement and behavioral change with digital health interventions. J Med Internet Res.

[23] Szeto K, Arnold J, Singh B, Gower B, Simpson CEM, Maher C (2023). Interventions using wearable activity trackers to improve patient physical activity and other outcomes in adults who are hospitalized: a systematic review and meta-analysis. JAMA Netw Open.

[24] Daryabeygi-Khotbehsara R, Shariful Islam SM, Dunstan D, McVicar J, Abdelrazek M, Maddison R (2021). Smartphone-based interventions to reduce sedentary behavior and promote physical activity using integrated dynamic models: systematic review. J Med Internet Res.

[25] Yerrakalva D, Yerrakalva D, Hajna S, Griffin S (2019). Effects of mobile health app interventions on sedentary time, physical activity, and fitness in older adults: systematic review and meta-analysis. J Med Internet Res.

[26] Moher D, Liberati A, Tetzlaff J, Altman DG, PRISMA Group (2009). Preferred reporting items for systematic reviews and meta-analyses: the PRISMA statement. BMJ.

[27] (2024). Noncommunicable diseases. World Health Organization.

[28] Castro EA, Júdice PB, Silva AM, Teixeira PJ, Benito PJ (2017). Sedentary behavior and compensatory mechanisms in response to different doses of exercise-a randomized controlled trial in overweight and obese adults. Eur J Clin Nutr.

[29] Tremblay MS, Aubert S, Barnes JD (2017). Sedentary behavior research network (SBRN)—terminology consensus project process and outcome. Int J Behav Nutr Phys Act.

[30] Higgins J, Thomas J (2023). Cochrane handbook for systematic reviews of interventions version 64.

[31] Dettori JR, Norvell DC, Chapman JR (2022). Fixed-effect vs random-effects models for meta-analysis: 3 points to consider. Global Spine J.

[32] Cohen J (1988). Statistical Power Analysis for the Behavioral Sciences.

[33] Higgins JPTS (2003). Measuring inconsistency in meta-analyses. BMJ.

[34] Ashizawa R, Honda H, Take K (2022). Approaches to promote reduction in sedentary behavior in patients with minor ischemic stroke: a randomized controlled trial. Arch Phys Med Rehabil.

[35] Ashizawa R, Honda H, Take K, Yoshizawa K, Kameyama Y, Yoshimoto Y (2023). Effects on sedentary behaviour of an approach to reduce sedentary behaviour in patients with minor ischaemic stroke: a randomised controlled trial. Clin Rehabil.

[36] Thomsen T, Aadahl M, Beyer N (2020). Sustained long‐term efficacy of motivational counseling and text message reminders on daily sitting time in patients with rheumatoid arthritis: long‐term follow‐up of a randomized, parallel‐group trial. Arthritis Care Res (Hoboken).

[37] Thomsen T, Aadahl M, Beyer N (2017). The efficacy of motivational counselling and SMS reminders on daily sitting time in patients with rheumatoid arthritis: a randomised controlled trial. Ann Rheum Dis.

[38] Sjöros T, Laine S, Garthwaite T (2023). Reducing sedentary time and whole-body insulin sensitivity in metabolic syndrome: a 6-month randomized controlled trial. Med Sci Sports Exerc.

[39] Norha J, Sjöros T, Garthwaite T (2023). Effects of reducing sedentary behavior on cardiorespiratory fitness in adults with metabolic syndrome: a 6-month RCT. Scand J Med Sci Sports.

[40] Sjöros T, Laine S, Garthwaite T (2023). The effects of a 6-month intervention aimed to reduce sedentary time on skeletal muscle insulin sensitivity: a randomized controlled trial. Am J Physiol Endocrinol Metab.

[41] den Uijl I, van den Berg-Emons RJG, Sunamura M (2023). Effects of a dedicated cardiac rehabilitation program for patients with obesity on body weight, physical activity, sedentary behavior, and physical fitness: the OPTICARE XL randomized controlled trial. Phys Ther.

[42] Lakerveld J, Bot SDM, van der Ploeg HP, Nijpels G (2013). The effects of a lifestyle intervention on leisure-time sedentary behaviors in adults at risk: the Hoorn Prevention Study, a randomized controlled trial. Prev Med.

[43] van Bakel BMA, Kroesen SH, Bakker EA (2023). Effectiveness of an intervention to reduce sedentary behaviour as a personalised secondary prevention strategy for patients with coronary artery disease: main outcomes of the SIT LESS randomised clinical trial. Int J Behav Nutr Phys Act.

[44] Bossen D, Veenhof C, Van Beek KE, Spreeuwenberg PM, Dekker J, De Bakker DH (2013). Effectiveness of a web-based physical activity intervention in patients with knee and/or hip osteoarthritis: randomized controlled trial. J Med Internet Res.

[45] Lynch BM, Courneya KS, Sethi P, Patrao TA, Hawkes AL (2014). A randomized controlled trial of a multiple health behavior change intervention delivered to colorectal cancer survivors: effects on sedentary behavior. Cancer.

[46] Lynch BM, Nguyen NH, Moore MM (2019). A randomized controlled trial of a wearable technology‐based intervention for increasing moderate to vigorous physical activity and reducing sedentary behavior in breast cancer survivors: the ACTIVATE Trial. Cancer.

[47] Maxwell-Smith C, Hince D, Cohen PA (2019). A randomized controlled trial of WATAAP to promote physical activity in colorectal and endometrial cancer survivors. Psychooncology.

[48] Patterson K, Davey R, Keegan R (2023). Testing the effect of a smartphone app on hospital admissions and sedentary behavior in cardiac rehabilitation participants: ToDo-CR randomized controlled trial. JMIR Mhealth Uhealth.

[49] Poppe L, De Bourdeaudhuij I, Verloigne M (2019). Efficacy of a self-regulation–based electronic and mobile health intervention targeting an active lifestyle in adults having type 2 diabetes and in adults aged 50 years or older: two randomized controlled trials. J Med Internet Res.

[50] Zheng C, Gill JMR, Sun FH (2023). Effects of increasing light versus moderate-to-vigorous physical activity on cardiometabolic health in Chinese adults with obesity. J Sports Sci.

[51] Pinto B, Dunsiger S, Stein K (2017). Does a peer-led exercise intervention affect sedentary behavior among breast cancer survivors?. Psychooncology.

[52] Biddle SJH, Edwardson CL, Wilmot EG (2015). A randomised controlled trial to reduce sedentary time in young adults at risk of type 2 diabetes mellitus: Project STAND (sedentary time and diabetes). PLoS ONE.

[53] Li LC, Feehan LM, Xie H (2020). Effects of a 12-week multifaceted wearable-based program for people with knee osteoarthritis: randomized controlled trial. JMIR Mhealth Uhealth.

[54] Li LC, Feehan LM, Xie H (2020). Efficacy of a physical activity counseling program with use of a wearable tracker in people with inflammatory arthritis: a randomized controlled trial. Arthritis Care Res (Hoboken).

[55] Devi R, Powell J, Singh S (2014). A web-based program improves physical activity outcomes in a primary care angina population: randomized controlled trial. J Med Internet Res.

[56] Su JJ, Yu DSF (2021). Effects of a nurse-led eHealth cardiac rehabilitation programme on health outcomes of patients with coronary heart disease: a randomised controlled trial. Int J Nurs Stud.

[57] Alghafri TS, Alharthi SM, Al-Farsi Y (2018). “MOVEdiabetes”: a cluster randomized controlled trial to increase physical activity in adults with type 2 diabetes in primary health in Oman. BMJ Open Diabetes Res Care.

[58] De Greef KP, Deforche BI, Ruige JB (2011). The effects of a pedometer-based behavioral modification program with telephone support on physical activity and sedentary behavior in type 2 diabetes patients. Patient Educ Couns.

[59] Garthwaite T, Sjöros T, Laine S (2022). Effects of reduced sedentary time on cardiometabolic health in adults with metabolic syndrome: a three-month randomized controlled trial. J Sci Med Sport.

[60] Gill DP, Blunt W, Boa Sorte Silva NC, Stiller-Moldovan C, Zou GY, Petrella RJ (2019). The HealtheSteps. BMC Public Health.

[61] Ter Hoeve N, Sunamura M, Stam HJ (2018). Effects of two behavioral cardiac rehabilitation interventions on physical activity: a randomized controlled trial. Int J Cardiol.

[62] Ormel HL, van der Schoot GGF, Westerink NDL, Sluiter WJ, Gietema JA, Walenkamp AME (2018). Self-monitoring physical activity with a smartphone application in cancer patients: a randomized feasibility study (SMART-trial). Support Care Cancer.

[63] Valle CG, Diamond MA, Heiling HM (2023). Effect of an mHealth intervention on physical activity outcomes among young adult cancer survivors: the IMPACT randomized controlled trial. Cancer.

[64] Brocklebank LA, Falconer CL, Page AS, Perry R, Cooper AR (2015). Accelerometer-measured sedentary time and cardiometabolic biomarkers: a systematic review. Prev Med.

[65] Priscila M, Vera Z, Élvio RG, Bruce J, Adilson M, Adilson M, Élvio RG (2021). Sedentary Behaviour.

[66] Gill DP, Blunt W, Bartol C (2017). HealtheSteps. BMC Public Health.

[67] Katzmarzyk PT, Ross R, Blair SN, Després JP (2020). Should we target increased physical activity or less sedentary behavior in the battle against cardiovascular disease risk development?. Atherosclerosis.

[68] Jandoo T (2020). WHO guidance for digital health: what it means for researchers. Digit Health.

[69] Stephenson A, McDonough SM, Murphy MH, Nugent CD, Mair JL (2017). Using computer, mobile and wearable technology enhanced interventions to reduce sedentary behaviour: a systematic review and meta-analysis. Int J Behav Nutr Phys Act.

[70] Gardner B, Smith L, Lorencatto F, Hamer M, Biddle SJH (2016). How to reduce sitting time? A review of behaviour change strategies used in sedentary behaviour reduction interventions among adults. Health Psychol Rev.

[71] Li C, Chen X, Bi X (2021). Wearable activity trackers for promoting physical activity: a systematic meta-analytic review. Int J Med Inform.

[72] Schepens Niemiec SL, Cariño B, Chatfield AJ, Quan K (2022). mHealth-supported interventions with potential to address sedentary behavior in older adults: a scoping review. J Aging Phys Act.

[73] Dong Q, Liu T, Liu R, Yang H, Liu C (2023). Effectiveness of digital health literacy interventions in older adults: single-arm meta-analysis. J Med Internet Res.

[74] Maddison R, Rawstorn JC, Stewart RAH (2019). Effects and costs of real-time cardiac telerehabilitation: randomised controlled non-inferiority trial. Heart.

[75] Motl RW, Sasaki JE, Cederberg KL, Jeng B (2019). Social-cognitive theory variables as correlates of sedentary behavior in multiple sclerosis: preliminary evidence. Disabil Health J.

[76] Martin A, Fitzsimons C, Jepson R (2015). Interventions with potential to reduce sedentary time in adults: systematic review and meta-analysis. Br J Sports Med.

[77] Buffey AJ, Herring MP, Langley CK, Donnelly AE, Carson BP (2022). The acute effects of interrupting prolonged sitting time in adults with standing and light-intensity walking on biomarkers of cardiometabolic health in adults: a systematic review and meta-analysis. Sports Med.

[78] Dempsey PC, Friedenreich CM, Leitzmann MF (2021). Global public health guidelines on physical activity and sedentary behavior for people living with chronic conditions: a call to action. J Phys Act Health.

[79] Dempsey PC, Larsen RN, Dunstan DW, Owen N, Kingwell BA (2018). Sitting less and moving more: implications for hypertension. Hypertension.

[80] Hibbing PR, Bellettiere J, Carlson JA (2022). Sedentary profiles: a new perspective on accumulation patterns in sedentary behavior. Med Sci Sports Exerc.

