# Supplementary material for: Effectiveness of Digital Health Interventions on Sedentary Behavior Among Patients With Chronic Diseases: Systematic Review and Meta-Analysis
Source: JMIR Mhealth Uhealth. 2025 Jun 24;13:e59943. doi: 10.2196/59943 (PMC12212891; doi:10.2196/59943)
Supplement: Multimedia Appendix 1 [file mhealth-v13-e59943-s001.docx]

**Search strategy**

1. **Pubmed**

| Search number | Query | Filters | Results |
| --- | --- | --- | --- |
| 7 | (("2000/01/01"[Date - Publication] : "2023/10/31"[Date - Publication])) AND ((((((((((((((Sedentary Behavior[MeSH Terms]) OR (Sedentary Lifestyle)) OR (Sedentary Time)) OR (Sitting time)) OR (Prolonged sitting time)) OR (Sedentary behavio*)) OR (Behavio*, Sedentary)) OR (Lifestyle, Sedentary)) OR (Time, Sedentary)) OR (Sitting pattern)) OR (sedentary pattern)) OR (Stationary behavio*))) AND (((((((((((((((((((((((((((((((((((((((digital technology[MeSH Terms]) OR (Digital health) OR (digital device) OR (Telemedicine) OR (Telerehabilitation)) OR (Telehealth)) OR (Telemonitor*)) OR (digital technology)) OR (Technology)) OR (Telephone)) OR (mobile phone)) OR (smartphone)) OR (mobile technology)) OR (phone-based)) OR (phone based)) OR (cell phone)) OR (electronic device)) OR (mobile device)) OR (monitor*)) OR (e-health)) OR (ehealth)) OR (electronic health)) OR (m-health)) OR (mhealth)) OR (mobile health)) OR (digital app)) OR (electronic app)) OR (artificial intelligen*)) OR (AI)) OR (immersive media)) OR (augment reality)) OR (ar)) OR (virtual reality)) OR (vr)) OR (mixed reality)) OR (mr)) OR (digital screen*)) OR (robot*)) OR (Internet)) OR (web-based)) OR (online)) OR (wearable*)) OR (wearable electronic devices)))) | Randomized Controlled Trial, English, Adult: 19+ years | 1,346 |
| 6 | (("2000/01/01"[Date - Publication] : "2023/10/31"[Date - Publication])) AND ((((((((((((((Sedentary Behavior[MeSH Terms]) OR (Sedentary Lifestyle)) OR (Sedentary Time)) OR (Sitting time)) OR (Prolonged sitting time)) OR (Sedentary behavio*)) OR (Behavio*, Sedentary)) OR (Lifestyle, Sedentary)) OR (Time, Sedentary)) OR (Sitting pattern)) OR (sedentary pattern)) OR (Stationary behavio*))) AND (((((((((((((((((((((((((((((((((((((((digital technology[MeSH Terms]) OR (Digital health) OR (digital device) OR (Telemedicine) OR (Telerehabilitation)) OR (Telehealth)) OR (Telemonitor*)) OR (digital technology)) OR (Technology)) OR (Telephone)) OR (mobile phone)) OR (smartphone)) OR (mobile technology)) OR (phone-based)) OR (phone based)) OR (cell phone)) OR (electronic device)) OR (mobile device)) OR (monitor*)) OR (e-health)) OR (ehealth)) OR (electronic health)) OR (m-health)) OR (mhealth)) OR (mobile health)) OR (digital app)) OR (electronic app)) OR (artificial intelligen*)) OR (AI)) OR (immersive media)) OR (augment reality)) OR (ar)) OR (virtual reality)) OR (vr)) OR (mixed reality)) OR (mr)) OR (digital screen*)) OR (robot*)) OR (Internet)) OR (web-based)) OR (online)) OR (wearable*)) OR (wearable electronic devices)))) | Randomized Controlled Trial, English | 1,869 |
| 5 | (("2000/01/01"[Date - Publication] : "2023/10/31"[Date - Publication])) AND ((((((((((((((Sedentary Behavior[MeSH Terms]) OR (Sedentary Lifestyle)) OR (Sedentary Time)) OR (Sitting time)) OR (Prolonged sitting time)) OR (Sedentary behavio*)) OR (Behavio*, Sedentary)) OR (Lifestyle, Sedentary)) OR (Time, Sedentary)) OR (Sitting pattern)) OR (sedentary pattern)) OR (Stationary behavio*))) AND (((((((((((((((((((((((((((((((((((((((digital technology[MeSH Terms]) OR (Digital health) OR (digital device) OR (Telemedicine) OR (Telerehabilitation)) OR (Telehealth)) OR (Telemonitor*)) OR (digital technology)) OR (Technology)) OR (Telephone)) OR (mobile phone)) OR (smartphone)) OR (mobile technology)) OR (phone-based)) OR (phone based)) OR (cell phone)) OR (electronic device)) OR (mobile device)) OR (monitor*)) OR (e-health)) OR (ehealth)) OR (electronic health)) OR (m-health)) OR (mhealth)) OR (mobile health)) OR (digital app)) OR (electronic app)) OR (artificial intelligen*)) OR (AI)) OR (immersive media)) OR (augment reality)) OR (ar)) OR (virtual reality)) OR (vr)) OR (mixed reality)) OR (mr)) OR (digital screen*)) OR (robot*)) OR (Internet)) OR (web-based)) OR (online)) OR (wearable*)) OR (wearable electronic devices)))) | Randomized Controlled Trial | 1,877 |
| 4 | (("2000/01/01"[Date - Publication] : "2023/10/31"[Date - Publication])) AND ((((((((((((((Sedentary Behavior[MeSH Terms]) OR (Sedentary Lifestyle)) OR (Sedentary Time)) OR (Sitting time)) OR (Prolonged sitting time)) OR (Sedentary behavio*)) OR (Behavio*, Sedentary)) OR (Lifestyle, Sedentary)) OR (Time, Sedentary)) OR (Sitting pattern)) OR (sedentary pattern)) OR (Stationary behavio*))) AND (((((((((((((((((((((((((((((((((((((((digital technology[MeSH Terms]) OR (Digital health) OR (digital device) OR (Telemedicine) OR (Telerehabilitation)) OR (Telehealth)) OR (Telemonitor*)) OR (digital technology)) OR (Technology)) OR (Telephone)) OR (mobile phone)) OR (smartphone)) OR (mobile technology)) OR (phone-based)) OR (phone based)) OR (cell phone)) OR (electronic device)) OR (mobile device)) OR (monitor*)) OR (e-health)) OR (ehealth)) OR (electronic health)) OR (m-health)) OR (mhealth)) OR (mobile health)) OR (digital app)) OR (electronic app)) OR (artificial intelligen*)) OR (AI)) OR (immersive media)) OR (augment reality)) OR (ar)) OR (virtual reality)) OR (vr)) OR (mixed reality)) OR (mr)) OR (digital screen*)) OR (robot*)) OR (Internet)) OR (web-based)) OR (online)) OR (wearable*)) OR (wearable electronic devices)))) |  | 32,904 |
| 3 | (((((((((((((Sedentary Behavior[MeSH Terms]) OR (Sedentary Lifestyle)) OR (Sedentary Time)) OR (Sitting time)) OR (Prolonged sitting time)) OR (Sedentary behavio*)) OR (Behavio*, Sedentary)) OR (Lifestyle, Sedentary)) OR (Time, Sedentary)) OR (Sitting pattern)) OR (sedentary pattern)) OR (Stationary behavio*))) AND (((((((((((((((((((((((((((((((((((((((digital technology[MeSH Terms]) OR (Digital health) OR (digital device) OR (Telemedicine) OR (Telerehabilitation)) OR (Telehealth)) OR (Telemonitor*)) OR (digital technology)) OR (Technology)) OR (Telephone)) OR (mobile phone)) OR (smartphone)) OR (mobile technology)) OR (phone-based)) OR (phone based)) OR (cell phone)) OR (electronic device)) OR (mobile device)) OR (monitor*)) OR (e-health)) OR (ehealth)) OR (electronic health)) OR (m-health)) OR (mhealth)) OR (mobile health)) OR (digital app)) OR (electronic app)) OR (artificial intelligen*)) OR (AI)) OR (immersive media)) OR (augment reality)) OR (ar)) OR (virtual reality)) OR (vr)) OR (mixed reality)) OR (mr)) OR (digital screen*)) OR (robot*)) OR (Internet)) OR (web-based)) OR (online)) OR (wearable*)) OR (wearable electronic devices))) |  | 37,962 |
| 2 | ((((((((((((((((((((((((((((((((((((((digital technology[MeSH Terms]) OR (Digital health) OR (digital device) OR (Telemedicine) OR (Telerehabilitation)) OR (Telehealth)) OR (Telemonitor*)) OR (digital technology)) OR (Technology)) OR (Telephone)) OR (mobile phone)) OR (smartphone)) OR (mobile technology)) OR (phone-based)) OR (phone based)) OR (cell phone)) OR (electronic device)) OR (mobile device)) OR (monitor*)) OR (e-health)) OR (ehealth)) OR (electronic health)) OR (m-health)) OR (mhealth)) OR (mobile health)) OR (digital app)) OR (electronic app)) OR (artificial intelligen*)) OR (AI)) OR (immersive media)) OR (augment reality)) OR (ar)) OR (virtual reality)) OR (vr)) OR (mixed reality)) OR (mr)) OR (digital screen*)) OR (robot*)) OR (Internet)) OR (web-based)) OR (online)) OR (wearable*)) OR (wearable electronic devices)) |  | 6,687,741 |
| 1 | ((((((((((((Sedentary Behavior[MeSH Terms]) OR (Sedentary Lifestyle)) OR (Sedentary Time)) OR (Sitting time)) OR (Prolonged sitting time)) OR (Sedentary behavio*)) OR (Behavio*, Sedentary)) OR (Lifestyle, Sedentary)) OR (Time, Sedentary)) OR (Sitting pattern)) OR (sedentary pattern)) OR (Stationary behavio*)) |  | 51,110 |

1. **Web of science**

| # | Search Query | Results |
| --- | --- | --- |
| 1 | TS= (Sedentary Behavior OR Sedentary Lifestyle OR Sedentary Time OR Sitting time OR Prolonged sitting time OR Sedentary behavio* OR Behavio*, Sedentary OR Lifestyle, Sedentary OR Time, Sedentary OR Sitting pattern OR sedentary pattern OR Stationary behavio*) | 97134 |
| 2 | TS= (digital technology OR Digital health OR digital device OR Telemedicine OR Telerehabilitation OR Telehealth OR Telemonitor* OR digital technology OR Technology OR Telephone OR mobile phone OR smartphone OR mobile technology OR phone-based OR phone based OR cell phone OR electronic device OR mobile device OR monitor* OR e-health OR ehealth OR electronic health OR m-health OR mhealth OR mobile health OR digital app OR electronic app OR artificial intelligen* OR AI OR immersive media OR augment reality OR ar OR virtual reality OR vr OR mixed reality OR mr OR digital screen* OR robot* OR Internet OR web-based OR online OR wearable* OR Wearable Electronic Devices) | 6359143 |
| 3 | #2 AND #1 | 14999 |
| 4 | ((LA=(English)) AND LA=(English)) AND DOP=(2000-01-01/2023-10-31) | 52335914 |
| 5 | #3 AND #4 | 14057 |
| 6 | ((DT=(Article)) AND ALL=(Adults)) AND ALL=(Randomized Controlled Trial) | 56562 |
| 7 | #6 AND #5 | 426 |

1. **SCOPUS**

ALL("Sedentary Behavior") OR ALL("Sedentary Lifestyle") OR ALL(" Sedentary ( ( ALL ( "digital technology" ) OR ALL ( "digital health" ) OR ALL ( "digital device" ) OR ALL ( "telemedicine" ) OR ALL ( "telerehabilitation" ) OR ALL ( "telehealth" ) OR ALL ( "telemonitor*" ) OR ALL ( "digital technology" ) OR ALL ( "technology" ) OR ALL ( "telephone" ) OR ALL ( "mobile phone" ) OR ALL ( "smartphone" ) OR ALL ( "mobile technology" ) OR ALL ( "phone-based" ) OR ALL ( "phone based" ) OR ALL ( "cell phone" ) OR ALL ( "electronic device" ) OR ALL ( "mobile device" ) OR ALL ( "monitor*" ) OR ALL ( "e-health" ) OR ALL ( "ehealth" ) OR ALL ( "electronic health" ) OR ALL ( "m-health" ) OR ALL ( "mhealth" ) OR ALL ( "mobile health" ) OR ALL ( "digital app" ) OR ALL ( "electronic app" ) OR ALL ( "artificial intelligen*" ) OR ALL ( "ai" ) OR ALL ( "immersive media" ) OR ALL ( "augment reality" ) OR ALL ( "ar" ) OR ALL ( "virtual reality" ) OR ALL ( "vr" ) OR ALL ( "mixed reality" ) OR ALL ( "mr" ) OR ALL ( "digital screen*" ) OR ALL ( "robot*" ) OR ALL ( "internet" ) OR ALL ( "web-based" ) OR ALL ( "online" ) OR ALL ( "wearable*" ) OR ALL ( "wearable electronic devices" ) ) AND ( ALL ( "sedentary behavior" ) OR ALL ( "sedentary lifestyle" ) OR ALL ( " sedentary time" ) OR ALL ( "sitting time" ) OR ALL ( "prolonged sitting" ) OR ALL ( "sedentary behavio*" ) OR ALL ( " behavio*, sedentary" ) OR ALL ( " lifestyle, sedentary" ) OR ALL ( " time, sedentary" ) OR ALL ( "sitting pattern" ) OR ALL ( "sedentary pattern" ) OR ALL ( "stationary behavio*" ) ) AND PUBYEAR > 1999 AND PUBYEAR < 2024 ) AND ( TITLE-ABS-KEY ( randomized AND controlled AND trial ) ) AND NOT TITLE ( protocol ) AND ( LIMIT-TO ( PUBSTAGE , "final" ) ) AND ( LIMIT-TO ( DOCTYPE , "ar" ) ) AND ( LIMIT-TO ( LANGUAGE , "english" ) ) AND ( LIMIT-TO ( EXACTKEYWORD , "adult" ) )

1. **Embase**

| Query | Name | Last updated |
| --- | --- | --- |
| #10 | #9 AND 'randomized controlled trial'/de | 467 |
| #9 | (#4 OR #5 OR #6) AND [article]/lim AND [english]/lim AND [adult]/lim AND [young adult]/lim AND [middle aged]/lim AND [aged]/lim AND [very elderly]/lim AND [2000-2023]/py | 17,739 |
| #8 | #7 AND (2000:py OR 2001:py OR 2003:py OR 2004:py OR 2005:py OR 2006:py OR 2007:py OR 2008:py OR 2009:py OR 2010:py OR 2011:py OR 2012:py OR 2013:py OR 2014:py OR 2015:py OR 2016:py OR 2017:py OR 2018:py OR 2019:py OR 2020:py OR 2021:py OR 2022:py OR 2023:py) AND 'randomized controlled trial'/de AND ([adult]/lim OR [aged]/lim OR [very elderly]/lim) AND 'Article'/it | 164,077 |
| #7 | #4 OR #5 OR #6 | 10,171,372 |
| #6 | digital AND health OR (digital AND device) OR telemedicine OR telehealth OR telerehabilitation OR (digital AND technology) OR technology OR telephone OR (mobile AND phone) OR smartphone OR (mobile AND technology) OR 'phone based' OR (phone AND based) OR (cell AND phone) OR (electronic AND device) OR (mobile AND device) OR monitor* OR 'e health' OR ehealth OR (electronic AND health) OR 'm health' OR mhealth OR (mobile AND health) OR (digital AND app) OR (electronic AND app) OR (artificial AND intelligen*) OR ai OR (immersive AND media) OR (augment AND reality) OR ar OR (virtual AND reality) OR vr OR (mixed AND reality) OR mr OR (igital AND screen*) OR robot OR internet OR 'web based' OR online OR wearable* | 10,167,687 |
| #5 | 'telemedicine'/exp | 73,654 |
| #4 | 'digital technology'/exp | 4,921 |
| #3 | #1 OR #2 | 34,936 |
| #2 | 'sedentary behavior':ti,ab,kw OR 'sedentary lifestyle':ti,ab,kw OR 'sedentary time':ti,ab,kw OR 'sitting time':ti,ab,kw OR 'prolonged sitting':ti,ab,kw OR 'sedentary behavio*':ti,ab,kw OR 'behavio*, sedentary':ti,ab,kw OR 'lifestyle, sedentary':ti,ab,kw OR 'time, sedentary':ti,ab,kw OR 'sitting pattern':ti,ab,kw OR 'sedentary pattern':ti,ab,kw OR 'stationary behavio*':ti,ab,kw | 24,485 |
| #1 | 'sedentary lifestyle'/exp | 21,221 |

1. **CINAHL Complete**

| **#** | **Query** | **Limiters/Results** |
| --- | --- | --- |
| S9 | (S4 OR S5 OR S6) AND (S2 AND S7) | Limiters - Full Text; Publication Date: 20000101-20231031; English Language; Research Article; Randomized Controlled Trials; Age Groups: All Adult |
|  |  | 499 |
| S8 | (S4 OR S5 OR S6) AND (S2 AND S7) | 23,168 |
| S7 | S4 OR S5 OR S6 | 1,775,030 |
| S6 | TX ( mhealth or mobile health or m-health or mobile app or mobile application ) OR TX ( digital application or apps or digital uses or digital programs or digital application ) OR TX electronic app OR TX artificial intelligen* OR TX ( AI or immersive media ) OR TX digital screen* OR TX robot* OR TX ( Internet or web-based or online ) OR TX ( wearable* or Wearable Electronic Devices ) OR TX ( wearable technology or wearable devices or wearable sensors ) OR ( augment reality or AR ) OR ( virtual reality or VR or mixed reality or MR ) | 899,211 |
| S5 | TX smartphone OR TX mobile technology OR TX phone-based OR TX phone based OR TX cell phone OR TX electronic device OR TX mobile device OR TX monitor* OR TX e-health OR TX ( ehealth or telecare or telehealth ) OR TX electronic health OR TX mhealth | 658,758 |
| S4 | TX ( (MH "Digital Technology") OR (MH "Digital Health") ) OR TX digital technology OR TX digital health OR TX digital device OR TX Telemedicine OR TX Telerehabilitation OR TX Telehealth OR TX Telemonitor* OR TX digital technology OR TX Technology OR TX telephone OR TX mobile phone | 845,605 |
| S3 | (MH "Digital Technology") OR (MH "Digital Health") | 4,130 |
| S2 | TX ( (MM "Life Style, Sedentary+") OR "sedentary behavior" ) OR TX sedentary lifestyle OR TX sedentary time OR TX sedentary OR TX sitting time OR TX prolonged sitting OR TX Sedentary behavio* OR TX Stationary behavio* OR TX Lifestyle, Sedentary OR TX Time, Sedentary OR TX silting patterns OR TX sedentary pattern | 44,052 |
| S1 | (MM "Life Style, Sedentary+") OR "sedentary behavior" | 7068 |

1. **Cochrane**

| ID | Search | Hits |
| --- | --- | --- |
| #1 | MeSH descriptor: [Sedentary Behavior] this term only | 1609 |
| #2 | Sedentary Behavior OR Sedentary Lifestyle OR Sedentary Time OR Sitting time OR Prolonged sitting time OR Sedentary behavio* OR Behavio*, Sedentary OR Lifestyle, Sedentary OR Time, Sedentary OR Sitting pattern OR sedentary pattern OR Stationary behavio*:ti,ab,kw | 11919 |
| #3 | MeSH descriptor: [Digital Technology] this term only | 32 |
| #4 | MeSH descriptor: [Telemedicine] this term only | 4346 |
| #5 | digital technology OR Digital health OR digital device OR Telemedicine OR Telerehabilitation OR Telehealth OR Telemonitor* OR digital technology OR Technology OR Telephone OR mobile phone OR smartphone OR mobile technology OR phone-based OR phone based OR cell phone OR electronic device OR mobile device OR monitor* OR e-health OR ehealth OR electronic health OR m-health OR mhealth OR mobile health OR digital app OR electronic app OR artificial intelligen* OR AI OR immersive media OR augment reality OR ar OR virtual reality OR vr OR mixed reality OR mr OR digital screen* OR robot* OR Internet OR web-based OR online OR wearable* OR Wearable Electronic Devices:ti,ab,kw | 317026 |
| #6 | #1 OR #2 | 11919 |
| #7 | #3 OR #4 OR #5 | 317071 |
| #8 | #6 AND #7 | 3737 |
| #9 | English:la | 1191039 |
| #10 | (adults) | 142612 |
| #11 | (randomized controlled trial):ti,ab,kw | 742398 |
| #12 | #8 AND #9 AND #10 AND #11 | 465 |

1. **ACM Digital Library**

[[All: "sedentary behavior"] OR [All: "sedentary lifestyle"] OR [All: "sedentary time"] OR [All: "sitting time"] OR [All: "prolonged sitting time"] OR [All: "sedentary behavio*"] OR [All: "sedentary"] OR [All: lifestyle, sedentary] OR [All: time, sedentary] OR [All: "sitting pattern"] OR [All: "sedentary pattern"] OR [All: "stationary behavio*"]] AND [[All: "digital technology"] OR [All: "digital health"] OR [All: "digital device"] OR [All: "telemedicine"] OR [All: "telerehabilitation"] OR [All: "telehealth"] OR [All: "telemonitor*"] OR [All: "technology"] OR [All: "telephone"] OR [All: "mobile phone"] OR [All: "smartphone"] OR [All: "mobile"] OR [All: "phone-based"] OR [All: "phone based"] OR [All: "cell phone"] OR [All: "electronic device"] OR [All: "mobile device"] OR [All: "monitor*"] OR [All: "e-health"] OR [All: "ehealth"] OR [All: "electronic health"] OR [All: "m-health"] OR [All: "mhealth"] OR [All: "mobile health"] OR [All: digital app] OR [All: electronic app] OR [All: artificial intelligen*] OR [All: ai] OR [All: "immersive media"] OR [All: "augment reality"] OR [All: "ar"] OR [All: "virtual reality"] OR [All: "vr"] OR [All: "mixed reality"] OR [All: "mr"] OR [All: "digital screen*"] OR [All: "robot*"] OR [All: "internet"] OR [All: "web-based"] OR [All: "online"] OR [All: "wearable*"] OR [All: "wearable electronic devices"]] AND [Title: randomized controlled trial] AND [E-Publication Date: (01/01/2001 TO 10/31/2023)]

**Excluded studies**

| **Study and year** | **Country** | **Population** | **Reason for exclusion** | **Reference** |
| --- | --- | --- | --- | --- |
| Bughin et al, 2021 | France | Obesity | Interventions not target SB or PA | Bughin, F., Bui, G., Ayoub, B., Blervaque, L., Saey, D., Avignon, A., Brun, J. F., Molinari, N., Pomies, P., Mercier, J., Gouzi, F., & Hayot, M. (2021). Impact of a Mobile Telerehabilitation Solution on Metabolic Health Outcomes and Rehabilitation Adherence in Patients With Obesity: Randomized Controlled Trial. JMIR mHealth and uHealth, 9(12), e28242. https://doi.org/10.2196/28242 |
| Maddison et al, 2019 | New Zealand | CHD | Interventions not target SB or PA | Maddison, R., Rawstorn, J. C., Stewart, R. A. H., Benatar, J., Whittaker, R., Rolleston, A., Jiang, Y., Gao, L., Moodie, M., Warren, I., Meads, A., & Gant, N. (2019). Effects and costs of real-time cardiac telerehabilitation: randomised controlled non-inferiority trial. Heart (British Cardiac Society), 105(2), 122–129. https://doi.org/10.1136/heartjnl-2018-313189 |
| Ormel et al, 2018 | Netherlands | cancer | Interventions not target SB or PA, pilot study | Ormel, H. L., van der Schoot, G. G. F., Westerink, N. L., Sluiter, W. J., Gietema, J. A., & Walenkamp, A. M. E. (2018). Self-monitoring physical activity with a smartphone application in cancer patients: a randomized feasibility study (SMART-trial). Supportive care in cancer : official journal of the Multinational Association of Supportive Care in Cancer, 26(11), 3915–3923. https://doi.org/10.1007/s00520-018-4263-5 |
| Ter Hoeve et al, 2018 | Netherlands | ACS | Interventions not target SB or PA, or address SB without digital technology | Ter Hoeve, N., Sunamura, M., Stam, H. J., Boersma, E., Geleijnse, M. L., van Domburg, R. T., & van den Berg-Emons, R. J. G. (2018). Effects of two behavioral cardiac rehabilitation interventions on physical activity: A randomized controlled trial. International journal of cardiology, 255, 221–228. https://doi.org/10.1016/j.ijcard.2017.12.015 |
| Avila et al, 2018 | Belgium | CAD | Interventions not target SB or PA | Avila, A., Claes, J., Goetschalckx, K., Buys, R., Azzawi, M., Vanhees, L., & Cornelissen, V. (2018). Home-Based Rehabilitation With Telemonitoring Guidance for Patients With Coronary Artery Disease (Short-Term Results of the TRiCH Study): Randomized Controlled Trial. Journal of medical Internet research, 20(6), e225. https://doi.org/10.2196/jmir.9943 |
| Kambic et al, 2023 | Slovenia | CAD | Control group also used digital technologies | Kambic, T., Šarabon, N., Hadžić, V., & Lainscak, M. (2023). Physical activity and sedentary behaviour following combined aerobic and resistance training in coronary artery disease patients: A randomised controlled trial. International journal of cardiology, 370, 75–79. https://doi.org/10.1016/j.ijcard.2022.10.157 |
| Valle et al, 2022 | America | cancer | Control group also used digital technologies | Valle, C. G., Diamond, M. A., Heiling, H. M., Deal, A. M., Hales, D. P., Nezami, B. T., Pinto, B. M., LaRose, J. G., Rini, C. M., & Tate, D. F. (2023). Effect of an mHealth intervention on physical activity outcomes among young adult cancer survivors: The IMPACT randomized controlled trial. Cancer, 129(3), 461–472. https://doi.org/10.1002/cncr.34556 |
| Cheng et al, 2022 | New Zealand | COPD | Control group also used digital technologies | Cheng, S. W. M., Alison, J., Stamatakis, E., Dennis, S., McNamara, R., Spencer, L., & McKeough, Z. (2022). Six-week behaviour change intervention to reduce sedentary behaviour in people with chronic obstructive pulmonary disease: a randomised controlled trial. Thorax, 77(3), 231–238. https://doi.org/10.1136/thoraxjnl-2020-214885 |
| Júdice et al, 2020 | Portugal | T2DM | Only use wearable trackers in two groups | Júdice PB, Magalhães JP, Rosa GB, Correia IR, Ekelund U, Sardinha LB. Sedentary behavior compensation to 1-year exercise RCT in patients with type 2 diabetes. Transl Sports Med. 2020; 3: 154–163. Magalhães, J. P., Júdice, P. B., Ribeiro, R., Andrade, R., Raposo, J., Dores, H., Bicho, M., & Sardinha, L. B. (2019). Effectiveness of high-intensity interval training combined with resistance training versus continuous moderate-intensity training combined with resistance training in patients with type 2 diabetes: A one-year randomized controlled trial. Diabetes, obesity & metabolism, 21(3), 550–559. https://doi.org/10.1111/dom.13551 |
| Balducci et al, 2019 | Italian | T2DM | Only use wearable trackers in two groups | Balducci, S., D'Errico, V., Haxhi, J., Sacchetti, M., Orlando, G., Cardelli, P., Vitale, M., Bollanti, L., Conti, F., Zanuso, S., Lucisano, G., Nicolucci, A., Pugliese, G., & Italian Diabetes and Exercise Study 2 (IDES_2) Investigators (2019). Effect of a Behavioral Intervention Strategy on Sustained Change in Physical Activity and Sedentary Behavior in Patients With Type 2 Diabetes: The IDES_2 Randomized Clinical Trial. JAMA, 321(9), 880–890. https://doi.org/10.1001/jama.2019.0922 |
| Wootton et al, 2017 | Australia | COPD | Only use wearable trackers in two groups | Wootton, S. L., Hill, K., Alison, J. A., Ng, L. W. C., Jenkins, S., Eastwood, P. R., Hillman, D. R., Jenkins, C., Spencer, L., Cecins, N., Straker, L., & McKeough, Z. J. (2017). Effects of ground-based walking training on daily physical activity in people with COPD: A randomised controlled trial. Respiratory medicine, 132, 139–145. https://doi.org/10.1016/j.rmed.2017.10.008 |
