# Supplementary material for: Effectiveness of Digital Health Interventions on Sedentary Behavior Among Patients With Chronic Diseases: Systematic Review and Meta-Analysis
Source: JMIR Mhealth Uhealth. 2025 Jun 24;13:e59943. doi: 10.2196/59943 (PMC12212891; doi:10.2196/59943)
Supplement: Multimedia Appendix 2 [file mhealth-v13-e59943-s002.docx]

Table S1. Characteristics of selected RCTs.

| **Study** | **Country** | **Population** | **Characteristics** | **Intervention** | **Control** | **Intervention**  **duration** | **Type of digital technology** | **Outcome parameters** |
| --- | --- | --- | --- | --- | --- | --- | --- | --- |
| Zheng et al, 2023 | China | Obesity | MVPA:N=20; Drop-out:1(5%); Age:31.5±8.5yr; M/F:11/8 LPA: N=20; Drop-out:3(15%); Age:35.2±5.0yr; M/F:8/9 C:N=19; Drop-out:2(10.5%); Age:30.5±6.1yr; M/F:9/8 | ·MVPA: continuous moderate-intensity exercise such as brisk walking or running ·LPA: achieve goal steps with a normal walking speed | Maintain usual lifestyle | 3 months | ·Wearable tracker  ·Web system to confirm  adherence | ·Body composition,WC  ·ST and PA ·Blood glucose, HbA1c, TC,TG, HDL, LDL, C-peptide, CRP, endothelin 1 |
| van Bakel et al, 2023 | Netherlands | CAD | I: N=108; Drop-out:11(10%); Age:63±10yr; M/F:83/25 C:N=104; Drop-out:6(6%); Age:64±10yr; M/F:81/23 | ·Patient education and goal setting  ·Motivational interview  ·(tele)monitoring and vibrotactile feedback | Usual care | 3 months | ·Smartphone app ·Wearable tracker with vibrotactile feedback | ·ST, LPA, MVPA, step count  ·QoL, self-management competencies ·Recurrent cardiovascular events ·Process evaluation |
| Thomsen et al, 2020 | Denmark | RA | I: N=75; Drop-out:3(4%); Age:59.7±10.7yr;M/F:14/61 C:N=75; Drop-out:9(12%); Age:59.5±12.7yr;M/F:15/60 | ·Motivational counselling to set goals and make action plan of daily sitting ·Tailored text messages to remind of goals of SB | Maintain usual lifestyle | 22 months | Text messages | ·SB ·Pain,fatigue,physical function ·TC, TG, average glucose |
| Thomsen et al, 2017 | Denmark | RA | I: N=75; Drop-out:1(1.3%); Age:59.7±10.7yr; M/F:14/61 C:N=75; Drop-out:2(2.7%); Age:59.5±12.7yr; M/F:15/60 | ·Motivational counselling to set goals and make action plan of daily sitting ·Tailored text messages to remind of goals of SB | Maintain usual lifestyle | 4 months | Text messages | ·ST ·Pain, fatigue, physical function, self-efficacy, QoL, BP, blood lipids, HbA1c ·BMI, WC and WHR |
| Su et al, 2021 | China | CHD | I: N=73; Drop-out:7(9.6%); Age:55.5±7.3yr; M/F:62/11 C:N=73; Drop-out:15(20.5%); Age:56.0±7.0yr; M/F:60/13 | Nurse-led eHealth CR-lifestyle behaviors package: goal-seting and empowerment action phases | Usual care | 3 months | ·Web-based platform ·Wechat | ·PA(including ST)  ·Health-promoting lifestyle habits, smoking cessation, cardiac self-efficacy ·Health-related QoL, psychological wellbeing |
| Sjöros et al, 2023 | Finland | Obesity | I: N=33; Drop-out:1(3%); Age:59±6yr; M/F:13/20 C:N=31; Drop-out:3(9.7%); Age:57±8yr; M/F:14/17 | ·Personal counseling with physiotherapist ·Self-monitoring ·Provide support in goal achievement | Maintain usual lifestyle | 6 months | ·Smartphone app ·Wearable tracker ·Phone calls | ·Insulin sensitivity  ·Body mass, FFM, body fat, BMI, WC, HbA1c ·SB and PA, step counts |
| Poppe et al, 2019 | Netherlands | T2DM | I-PA: N=24; Drop-out: 11(45.8%); Age:62.9±7.16yr; M/F:17/7 I-SB: N=12; Drop-out: 2(16.7%); Age:58.9±9.52yr; M/F:8/4 C:N=18; Drop-out:1(5.6%); Age:64.9±8.62yr; M/F:9/9 | ·Action planning  ·Barrier identification and problem solving  ·Promoting self-monitoring  ·Social support  ·Review of behavioral goals | Wait-list control | 6 months | ·Web coaching ·Mobile app  ·Email | ·PA and SB ·Self-efficacy |
| Pinto et al, 2017 | America | Cancer | I: N=39; Drop-out:3(7.7%); Age:55.6±8.6yr;  C：N=37; Drop-out:6(16.2%); Age:55.6±10.9yr all females | ·PA counseling ·Behavior monitoring and regular feedback | Only equated frequency of contact | 6 months | ·Phone calls  ·Pedometer  ·Heart rate monitor | ·ST ·Fatigue ·QoL |
| Patterson et al, 2023 | Australia | CHD | I: N=60; Drop-out:11(18.3%); Age:61.1±10.1yr; M/F:48/12 C:N=60; Drop-out:4(6.7%); Age:64.1±9.9yr; M/F:45/15 | ·CR ·ToDo-CR program with SB monitoring, and personalized messages to encourage habits change | Usual care-CR | 12 months | ·Smartphone app  ·Wearable tracker | ·Hospital admissions and emergency presentations ·SB and PA ·App usability and engagement ·Economic evaluation |
| Maxwell et al, 2019 | Australia | Cancer | I: N=34; Drop-out:0; Age:65.3±7.4yr; M/F:13/21  C:N=34; Drop-out:1(2.9%); Age:62.9±8.4yr; M/F: 21/13 | ·Self-monitoring  ·Group sessions emphasizing on SB reduction  ·Supported information | Print materials | 3 months | ·Wearable tracker  ·Phone calls | MVPA, SB, BP, and BMI |
| Lynch et al, 2019 | Australia | Cancer | I: N=43; Drop-out:3(7.0%); Age:61.3±5.9yr;  C: N=40; Drop-out:0; Age:61.9±7yr; all females | ·Activity monitor coupled with a behavioral feedback  ·Goal-setting session ·Telephone-delivered health coaching sessions | Usual care | 3 months | ·Wearable tracker with inactivity alerts ·Phone calls | ·MVPA ·SB |
| Lynch et al, 2014 | Australia | Cancer | I: N=205; Drop-out:45(21.9%); Age:64.9±10.8yr; M/F:106/99 C:N=205; Drop-out:41(20%); Age:67.8±9.2yr; M/F:115/90 | Telephone delivered health coaching, handbook | Educational brochures and newsletter | 12 months | Phone calls | SB |
| Li et al, 2020(Dec) | Canada | OA | I: N=26; Drop-out:2(7.7%); Age:65.0±8.0yr; M/F:3/23 C:N=25; Drop-out:1(4%); Age:64.8±9.0yr; M/F:6/19 | ·In-person group education ·Self-monitoring  ·SB goal setting ·Physiotherapist counseling by phone | Wait-list control | 13 weeks | ·Web-based app connected to wearable tracker ·Phone calls | ·MVPA,steps,ST ·OA outcome ·Self-management behavior, motivation, depression, habitual behavior and adverse event |
| Li et al, 2020(Jul) | Canada | RA | I: N=59; Drop-out:3(5.1%); Age:53.5±14.7yr; M/F:8/51 C:N=59; Drop-out:2(3.4%); Age:53.1±12.6yr; M/F:5/54 | ·In-person group education ·Self-monitoring  ·SB goal setting ·Physiotherapist counseling by phone | Wait-list control | 9 weeks | ·Web-based app connected to wearable tracker ·Phone calls | ·MVPA,steps, ST ·Pain, fatigue, mood ·Self management capacity, and habitual behaviors |
| Lakerveld et al, 2013 | Netherlands | Obesity | I: N=314; Drop-out:72(22.9%); Age:43.6±5.1yr; M/F:136/178 C:N=308; Drop-out:41(19.5%);  Age:43.4±5.5yr; M/F:123/185 | Lifestyle counselling followed by phone, and practical coaching | Usual care-health brochures | 24 months | Phone calls | ST, television viewing, computer use, reading and other sedentary leisure time |
| Gill et al, 2019 | Canada | Mets | I: N=59; Drop-out:5(8.5%); Age:56.8±12.3yr; M/F:14/45 C:N=59; Drop-out:4(6.8%); Age:58.6±14.7yr; M/F:11/48 | HealtheSteps™ program ·Coach's prescriptions on PA  ·Customized health technology (eHealth) tools and resources | Usual care | 6 months | ·Phone calls  ·Smartphone app with a virtual coach  ·Web | ·Steps, PA and ST ·Eating habits ·Weight and body composition, BP and QoL |
| Garthwaite et al, 2022 | Finland | MetS | I: N=33; Drop-out:0;  Age:59±6yr; M/F:13/20 C:N=3; Drop-out:1(3.2%); Age:57±7yr; M/F:14/17 | ·Tailored personal counseling to sit less ·Self-monitoring ·Phone contacts and research center visit to receive support | Maintain usual lifestyle | 3 months | ·Smartphone app  ·Phone calls | ·SB, LPA, MVPA, standing time and steps ·Fasting insulin, HOMA-IR, HbA1c, TC, ALT, fasting glucose, FFM, AST, GGT and cholesterol |
| Devi et al, 2014 | England | Angina | I: N=48; Drop-out:7(14.6%); Age:66.3±8.4yr; M/F:34/14 C:N=46; Drop-out:3(6.5%); Age:66.2±10.6yr; M/F:36/10 | Web-based rehabilitation: setting/reviewing behavioral goals, self-monitoring, feedback on behavior, graded tasks, social reward, and reducing negative emotions | Usual care | 6 months | Web | ·Steps  ·Energy expenditure, SB, MPA,weight, BP, and body fat percentage ·Fat/fiber intake, anxiety/depression, self-efficacy, and QoL |
| den Uijl et al, 2023 | Netherlands | Obesity | I: N=102; Drop-out:6(5.9%); Age:59.0±10.0yr; M/F:68/34 C:N=99; Drop-out:8(8.1%); Age:59.2±8.8yr; M/F: 78/21 | ·Active lifestyle module: self-monitoring and goal setting ·Peer support by group chat on phone | Usual care-CR | 12 months | ·Wearable tracker  ·Smartphone app ·Phone group chat | ·QoL ·Body weight, PA,SB, and physical fitness |
| De Greef et al, 2011 | Belgium | T2DM | I: N=60; Drop-out:2(3.3%); Age:62±9yr; M/F:41/19 C:N=32; Drop-out:2(6.25%); Age:62±9yr; M/F: 22/10 | ·Face-to-face session with motivational interview  ·Self-monitoring ·Telephone support program | Usual care | 12 months | Phone calls | PA and SB |
| Bossen et al, 2013 | Netherlands | OA | I:N=100; Drop-out:24(24%); Age:61±5.9yr; M/F:40/60 C:N=99; Drop-out:26(26.3%); Age:63±5.4yr; M/F:30/69 | Join2move intervention: incorporates baseline test, goal setting, time-contingent PA objectives with automatic functions | Wait-list control | 12 months | ·Wearable tracker  ·Web-based automatic text messaging and emails | ·PA and SB ·Physical function ·Self-perceived effect ·Pain and fatigue |
| Castro et al, 2017 | Spain | Obesity | S:N=43; Drop-out:22(51.1%); Age:39.2±9.1yr E: N=51; Drop-out:24(47.1%); Age:40.6±6.6yr SE:N=46; Drop-out:22(47.8%); Age:37±7.5yr PA:N=40; Drop-out:23(57.5%); Age:41.1±5.9yr all completers: M/F:43/46 | S: scheduled exercises for strength training  E: endurance training  SE: combination of strength and endurance training | PA recommendations | 22 weeks | ·Electronic device  ·Wearable tracker | ·PA and SB ·Body composition: weight, height, fat, lean body mass, VO2max |
| Biddle et al, 2015 | UK | Obesity | I: N=94; Drop-out:30(31.9%); Age:32.4±5.4yr; M/F:28/66 C:N=93; Drop-out:25(26.9%); Age:33.3±5.8yr; M/F:31/62 | ·Group-based structured education workshop  ·Self-monitoring  ·Motivational phone call | Usual care with leaflet | 12 months | ·Wearable tracker  ·Phone calls | ·SB, PA ·Glucose control and insulin sensitivity ·TC, HDL,LDL, TG ·QoL, self-efficacy, anxiety and depression |
| Ashizawa et al, 2023 | Japan | Stroke | I: N=43; Drop-out:6(14.0%); Age:72±8.4yr; M/F:29/14 C:N=43; Drop-out:7(16.3%); Age:71.8±7.6yr; M/F:29/14 | ·During hospitalization: education on SB reduction, goal setting; self-monitoring with checklist. ·After discharge: self-monitor, phone call and stickers | ·During hospitalization: education on increasing PA, self-monitoring ·After hospital discharge: no | 6 months | Phone calls | ·SB,LPA, MVPA,steps, screen time  ·Self efficacy ·Depressive symptoms ·Sleep disorders |
| Ashizawa et al, 2022 | Japan | Stroke | I: N=31; Drop-out:5(16.1%); Age:72.3±8.9yr; M/F:19/12 C:N=30; Drop-out:4(13.3%); Age:70.3±7.7yr; M/F:21/9 | ·During hospitalization: education on SB reduction, goal setting; self-monitoring with checklist. ·After discharge: self-monitor, phone call and stickers | ·During hospitalization: education on increasing PA, self-monitoring ·After hospital discharge: no | 3 months | Phone calls | ·SB, LPA,MVPA, step count, screen time ·Depression status ·Sleep disorder |
| Alghafri et al, 2018 | Oman | T2DM | I: N=122; Drop-out:40(32.8%); Age:45.3±7.1yr; M/F:56/66 C:N=110; Drop-out:18(16.4%); Age:45.1±9.2yr; M/F:39/71 | ·Personalized face-to-face consultations ·Self- monitoring and feedback ·Monthly motivated messages | Usual care | 12 months | ·Smartphone app  ·Phone calls ·Text messages | ·PA, steps, ST ·BMI, HbA1c, BP and lipids |
| I: intervention group; C: control group; M: male, F: female; MetS: metabolic syndrome; T2DM: type 2 diabetes mellitus; HbA1c: hemoglobin A1c; SB: sedentary behavior; ST: sitting time; PA: physical activity; LPA: light physical activity; MVPA: moderate-to-vigorous physical activity; VO2 max: maximal aerobic exercise capacity; CHD: coronary heart disease; CAD: coronary artery disease; OA: osteoarthritis; RA: rheumatoid arthritis; SLE: systemic lupus erythematosus; CR: cardiac rehabilitation; BP: blood pressure; QoL: quality of life; FFM: fat free mass; HOMA-IR: homeostatic model assessment of insulin resistance; ALT: alanine aminotransferase; AST: aspartate aminotransferase; GGT: γ-glutamyltransferase; HDL: high-density lipoprotein; LDL: low-density lipoprotein; TC: total cholesterol; TG: triglycerides; I-PA: intervention targeting physical activity; I-SB: intervention targeting sedentary behavior; WC: waist circumference; CRP: C-reactive protein; WHR:waist–hip ratio. | | | | | | | | |
