# Supplementary figures and images for: Effectiveness of Digital Health Interventions on Sedentary Behavior Among Patients With Chronic Diseases: Systematic Review and Meta-Analysis
Source: JMIR Mhealth Uhealth. 2025 Jun 24;13:e59943. doi: 10.2196/59943 (PMC12212891; doi:10.2196/59943)

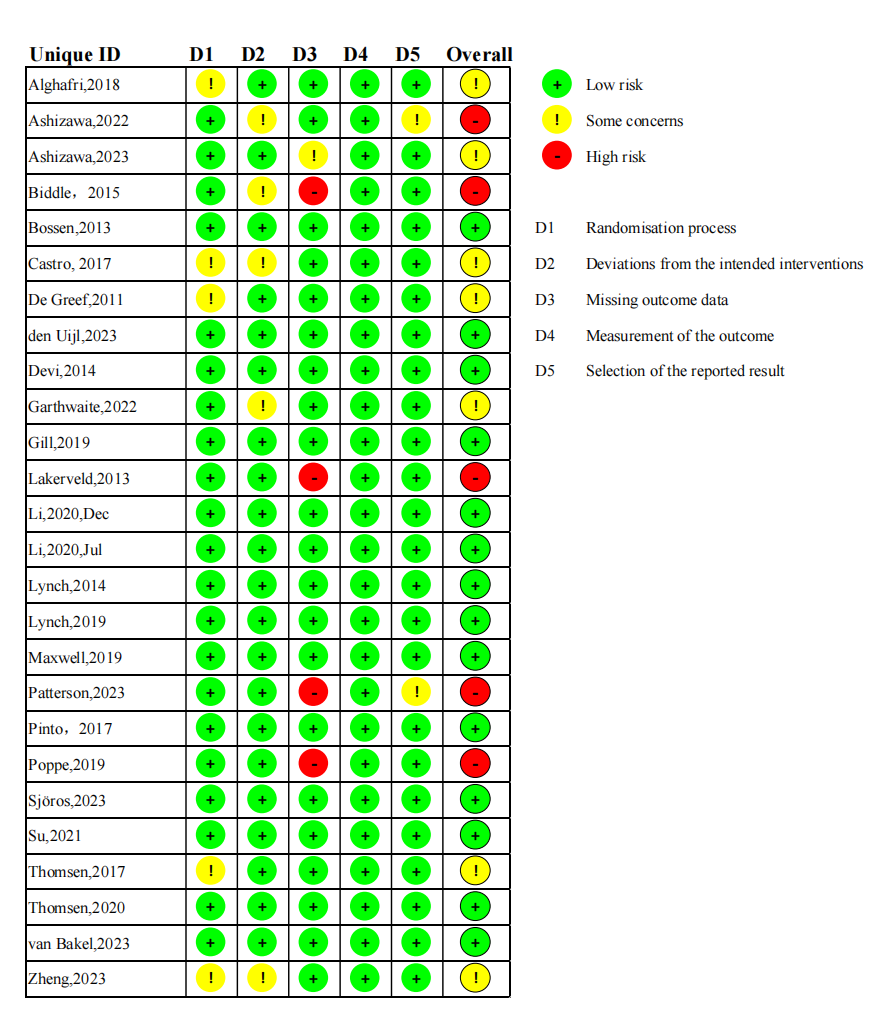


**Figure 1.** Risk of bias of the included study


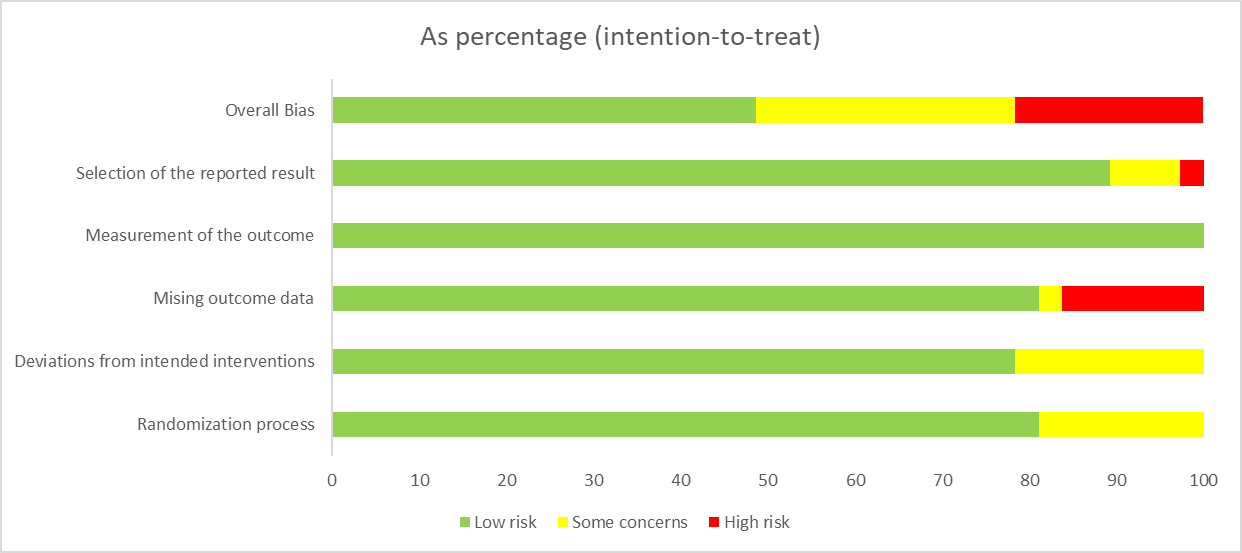


**Figure 2.** Summary of risk bias

Supplement: Multimedia Appendix 3 [file mhealth-v13-e59943-s003.docx]
