# Supplementary material for: Effectiveness of Digital Health Interventions on Sedentary Behavior Among Patients With Chronic Diseases: Systematic Review and Meta-Analysis
Source: JMIR Mhealth Uhealth. 2025 Jun 24;13:e59943. doi: 10.2196/59943 (PMC12212891; doi:10.2196/59943)
Supplement: Multimedia Appendix 4 [file mhealth-v13-e59943-s004.doc]

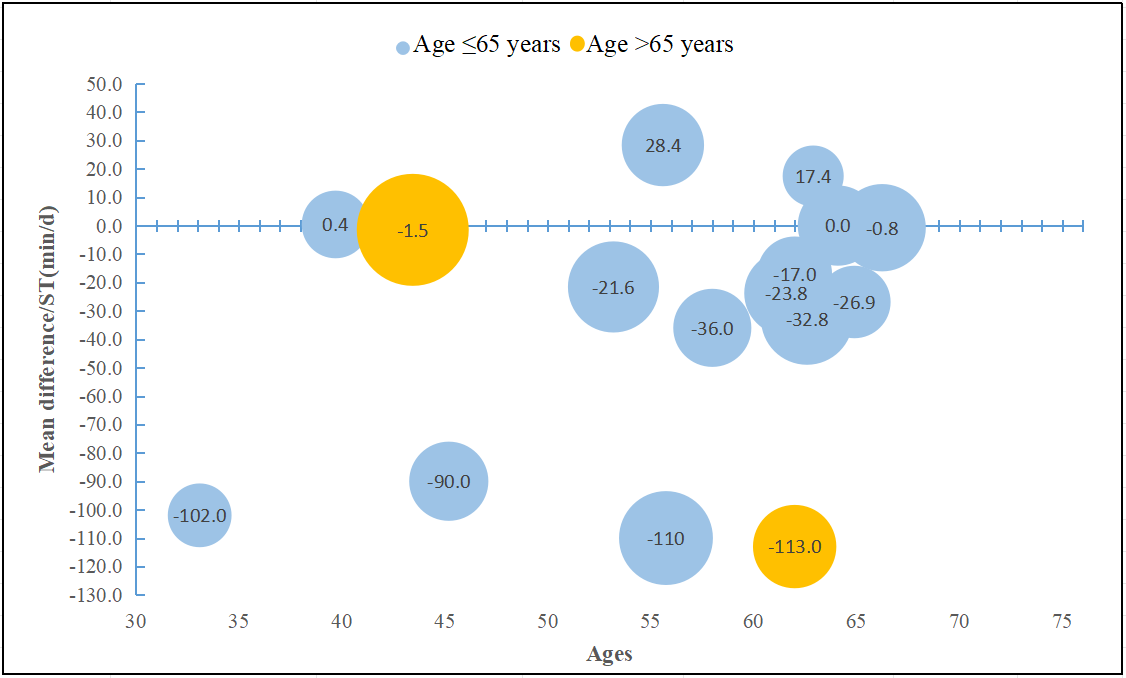


*ST, sitting time (min/d).

**Figure 1**. Grouped bubble plot of the study weight, mean difference of individual trial, and the mean age of the participants. Sixteen RCTs were included in the plot.
