# Supplementary material for: Effectiveness of Digital Health Interventions on Sedentary Behavior Among Patients With Chronic Diseases: Systematic Review and Meta-Analysis
Source: JMIR Mhealth Uhealth. 2025 Jun 24;13:e59943. doi: 10.2196/59943 (PMC12212891; doi:10.2196/59943)
Supplement: Multimedia Appendix 5 [file mhealth-v13-e59943-s005.docx]

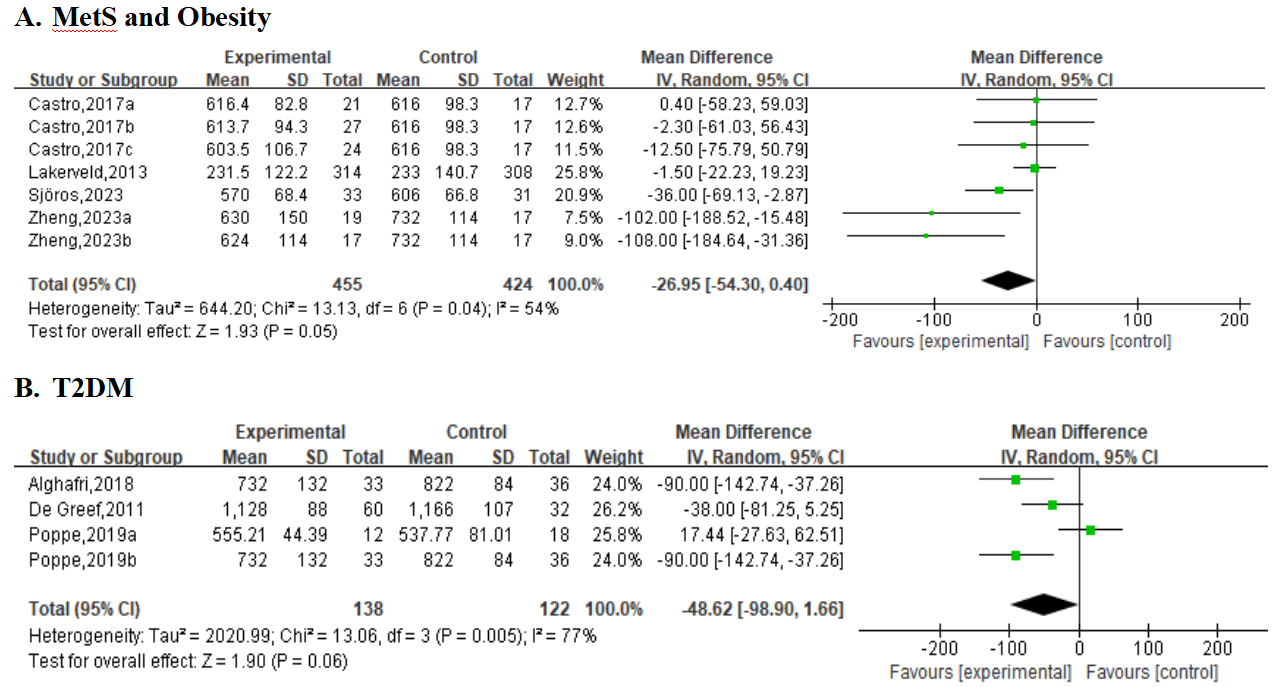


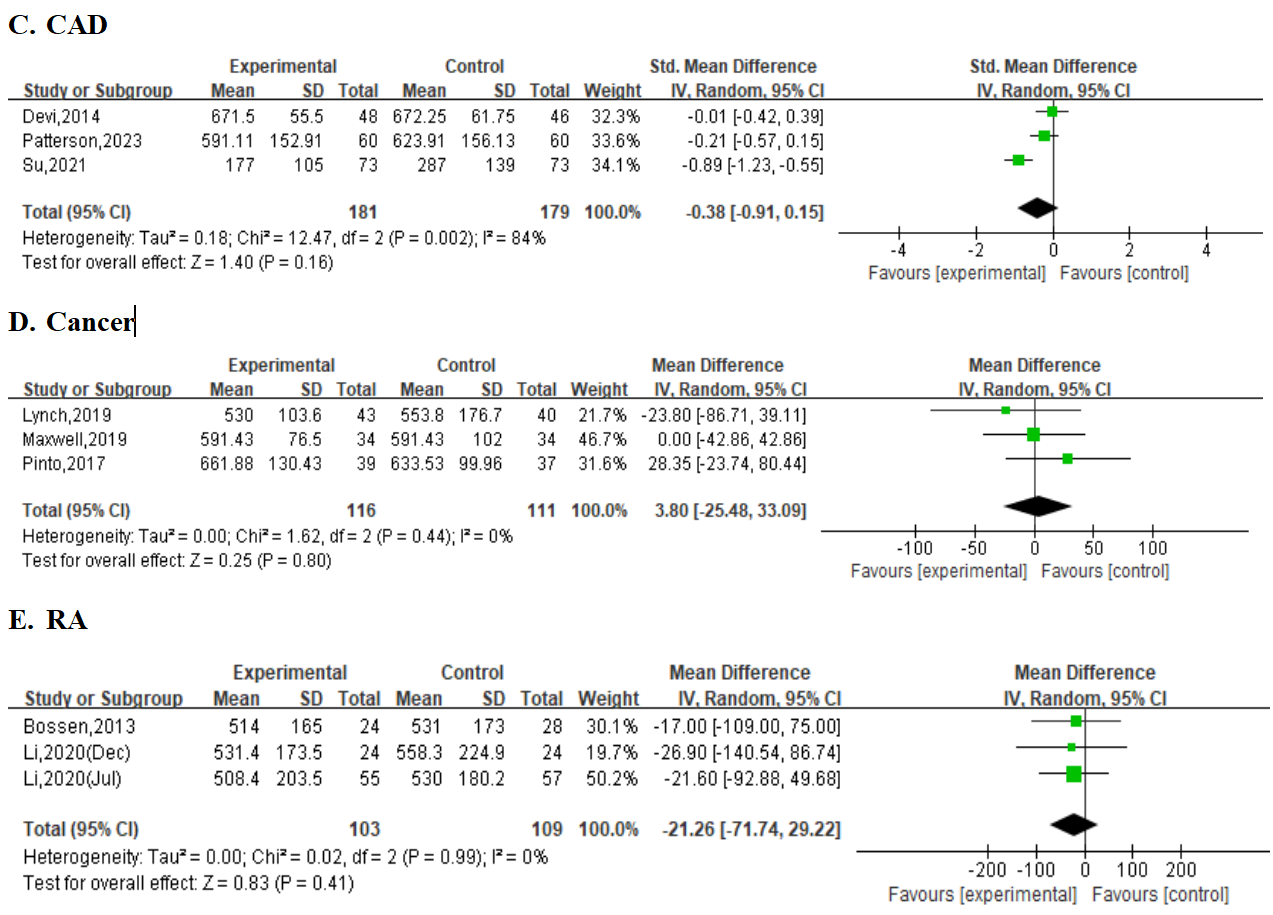


**Figure 1.** Subgroup analysis of different kinds of chronic diseases


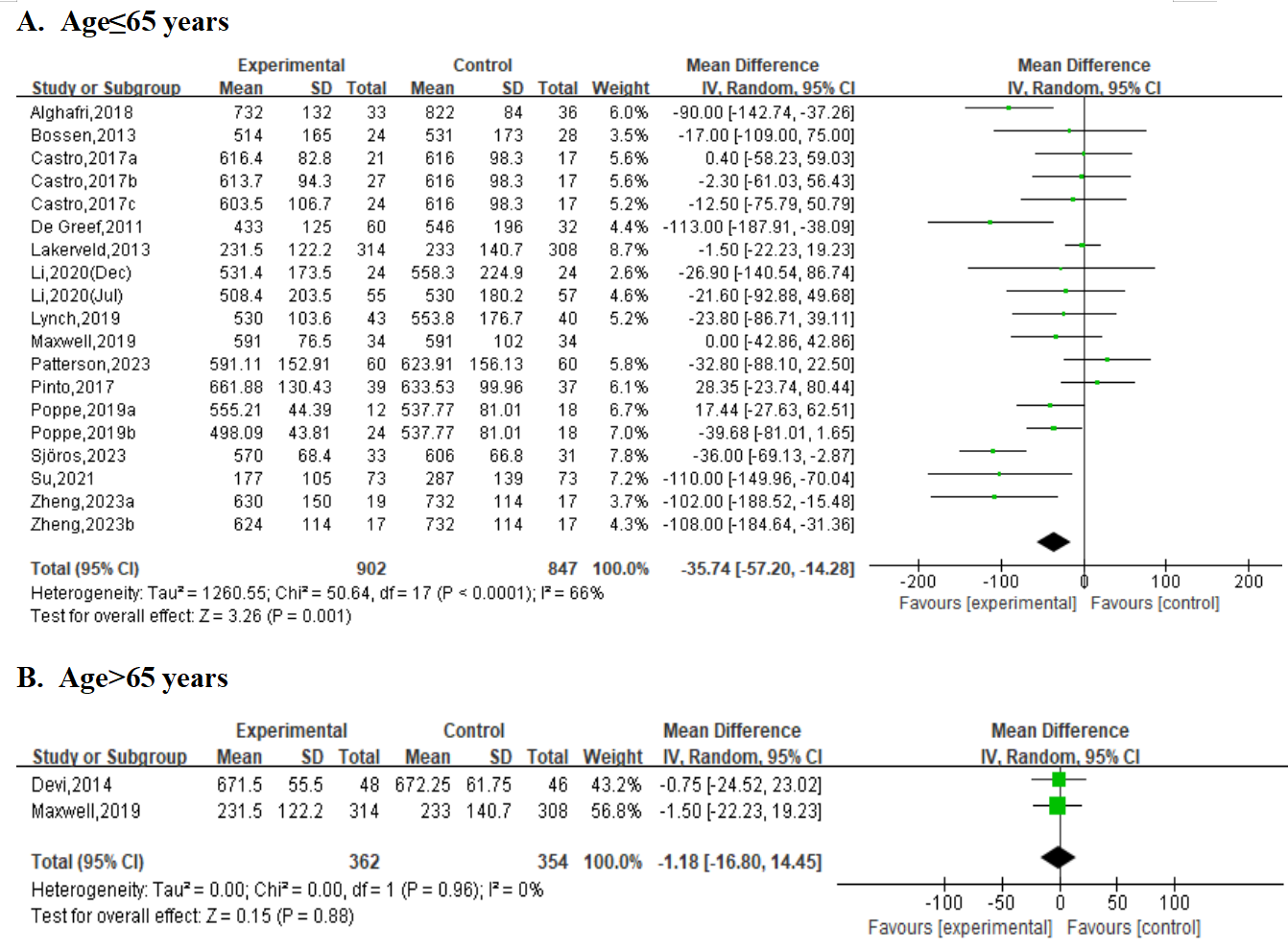


**Figure 2.** Subgroup analysis based on participants age (≤65 or >65 years old)


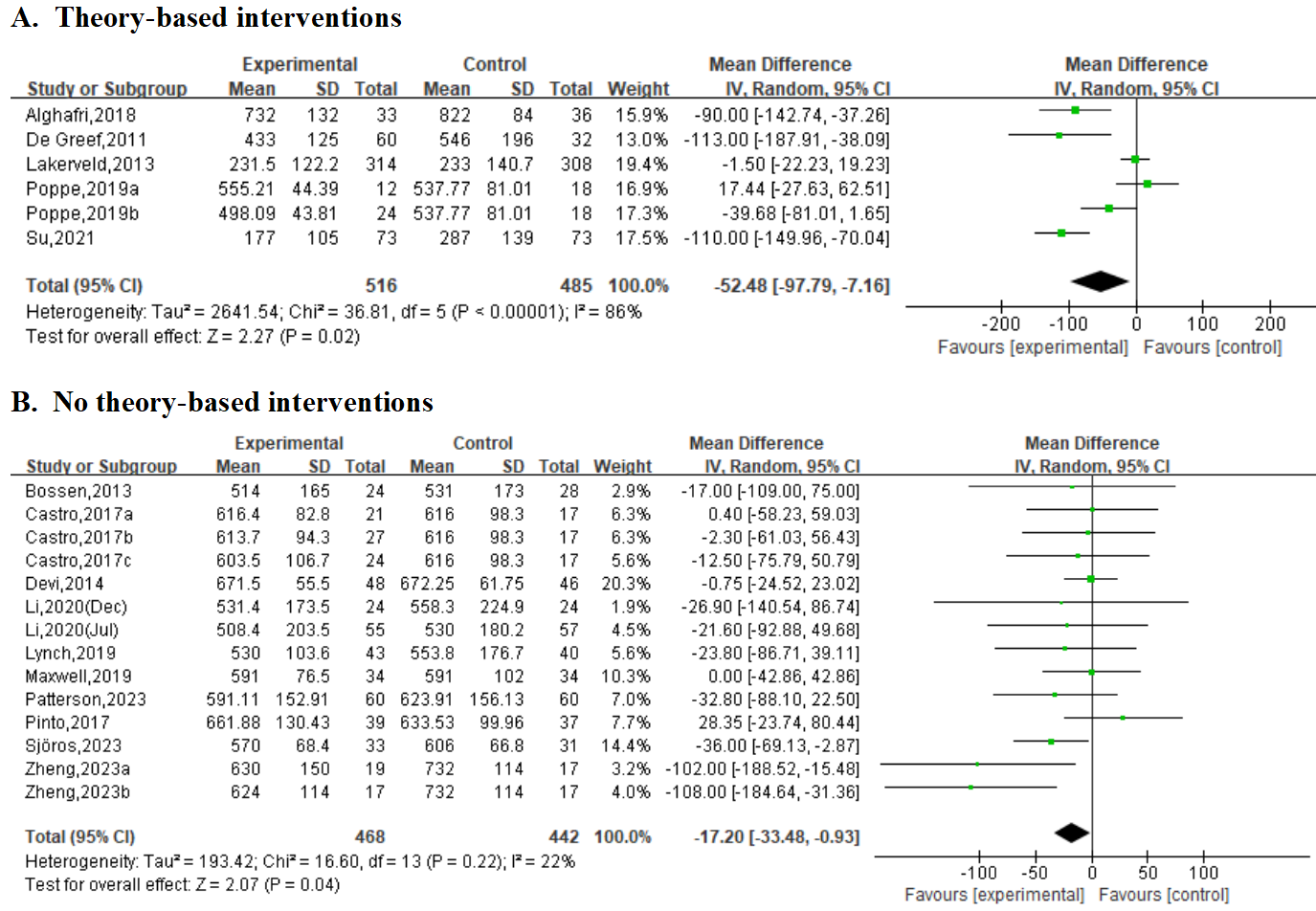


**Figure 3.** Subgroup analysis of interventions based on theory or not


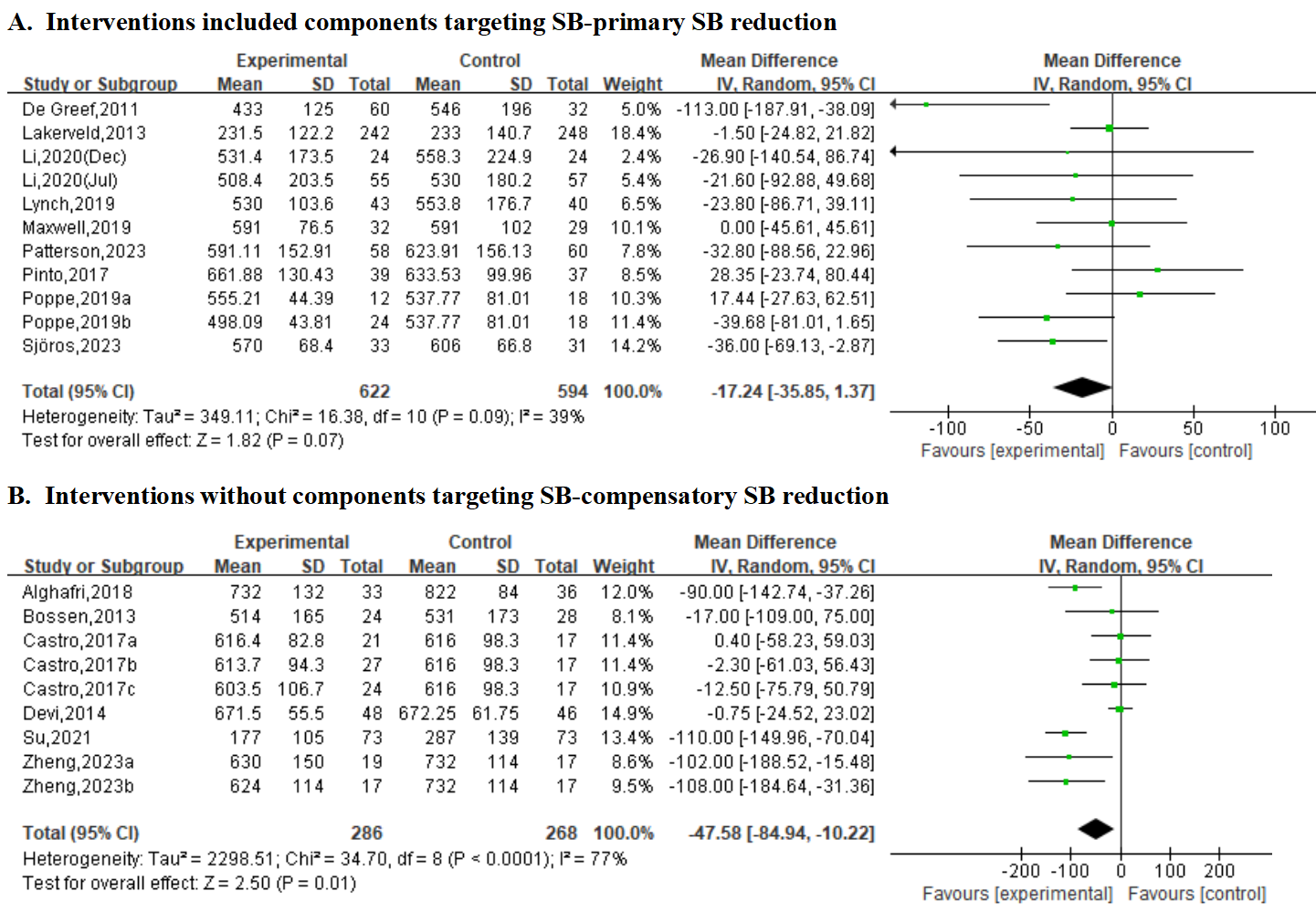


**Figure** **4.** Subgroup analysis of interventions with/without components targeting SB.


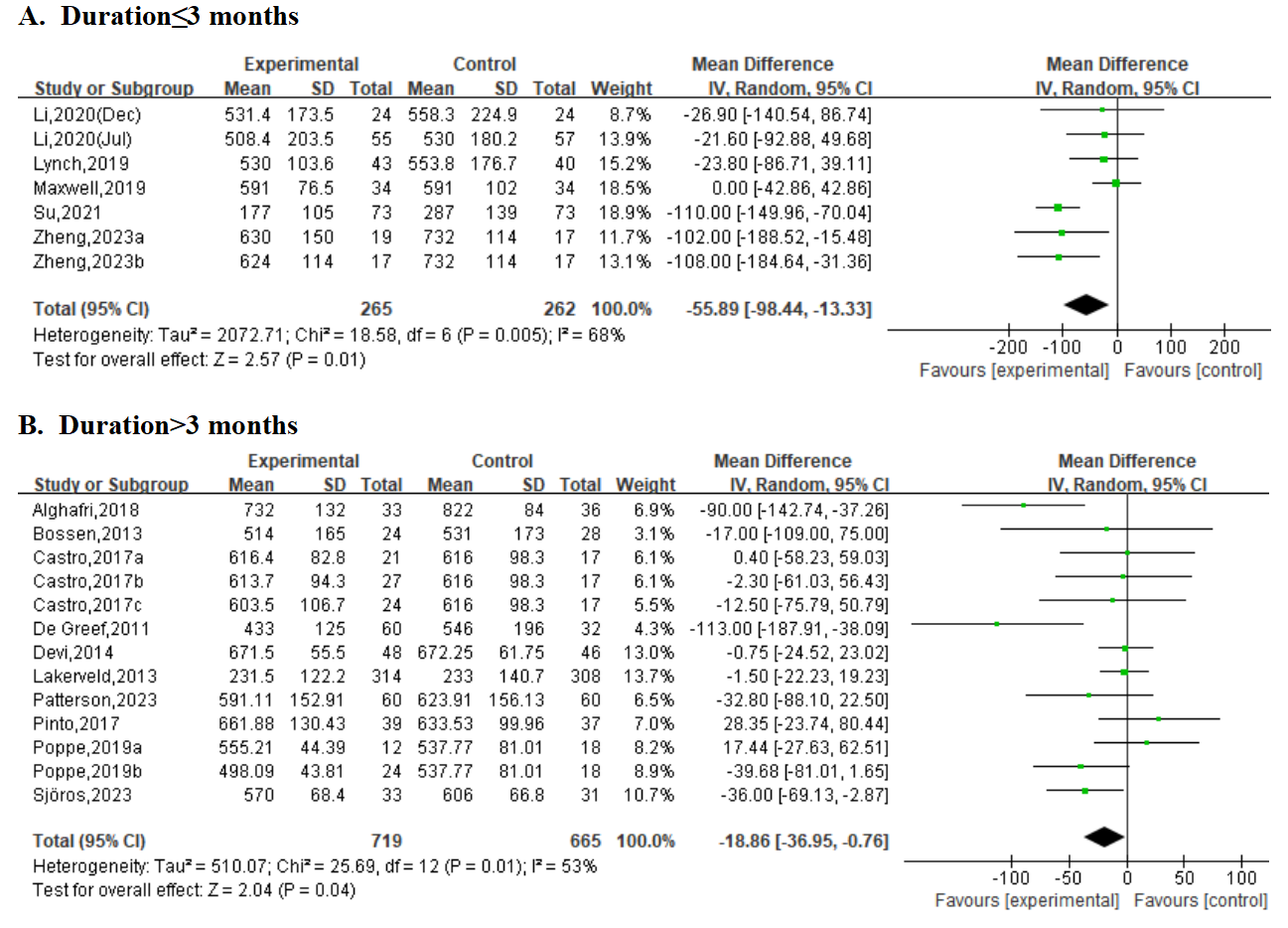


**Figure 5.** Subgroup analysis of different intervention duration (≤3months or >3months)


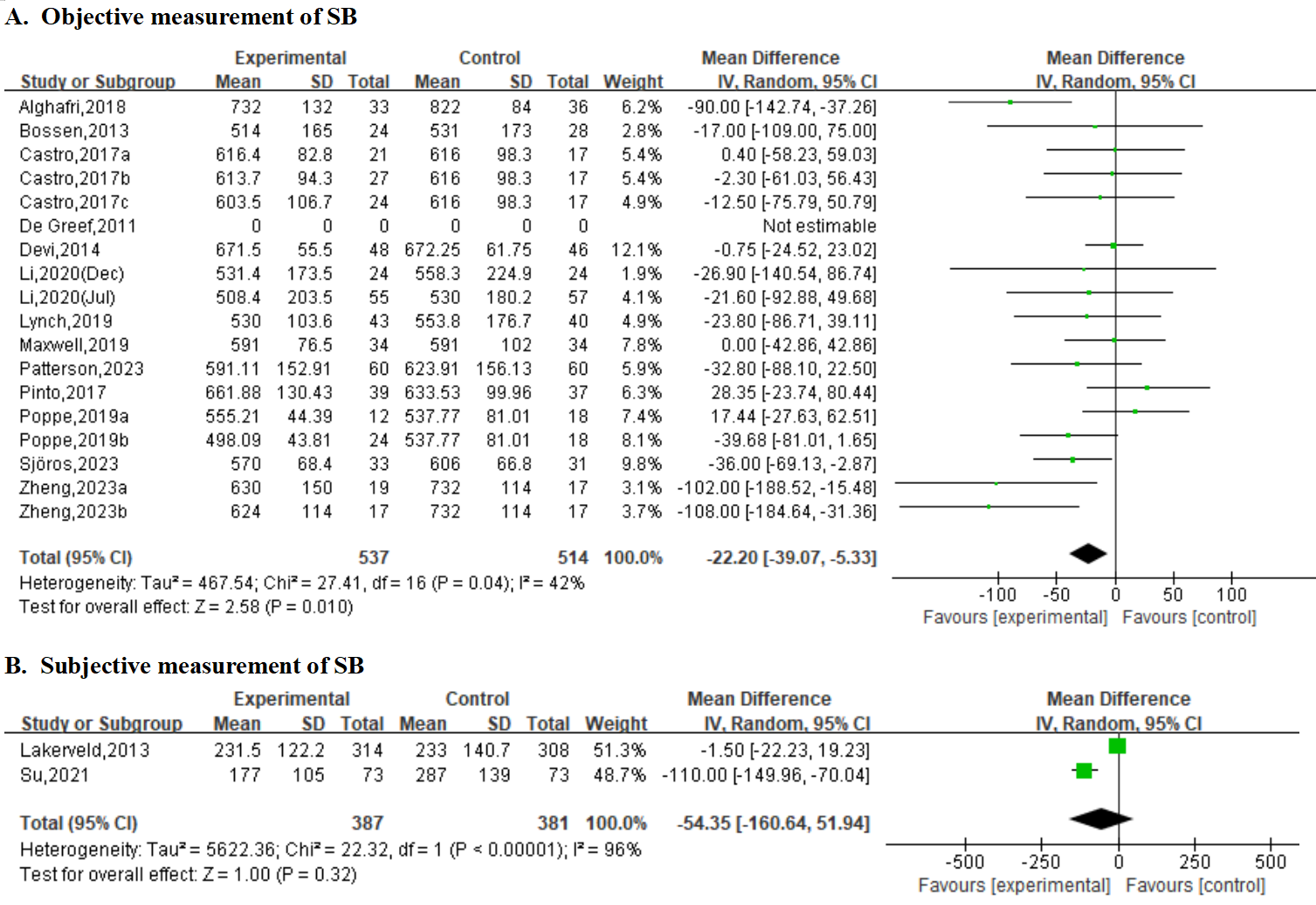


**Figure 6.** Subgroup analysis of objective or subjective measurement of SB
