# Supplementary material for: Effectiveness of Digital Health Interventions on Sedentary Behavior Among Patients With Chronic Diseases: Systematic Review and Meta-Analysis
Source: JMIR Mhealth Uhealth. 2025 Jun 24;13:e59943. doi: 10.2196/59943 (PMC12212891; doi:10.2196/59943)
Supplement: Checklist 1 [file mhealth-v13-e59943-s006.docx]

| **Section and Topic** | **Item #** | **Checklist item** | **Location where item is reported** |
| --- | --- | --- | --- |
| **TITLE** | | |  |
| Title | 1 | Identify the report as a systematic review. | “Effect of digital health interventions on sedentary behavior among patients with chronic diseases: a systematic review and meta-analysis” |
| **ABSTRACT** | | |  |
| Abstract | 2 | See the PRISMA 2020 for Abstracts checklist. | In this review, the authors summarize the objectives, eligibility criteria, databases searched, and methods to assess risk of bias and synthesize results, along with results of meta-analyses, indicating the number of studies and participants  included in each. See the details in the “Abstract” part. |
| **INTRODUCTION** | | |  |
| Rationale | 3 | Describe the rationale for the review in the context of existing knowledge. | The authors report the knowledge gap and limitations of the existing evidence base. See details in the “Introduction” part.  “Currently, many researchers have applied digital health interventions for SB reduction. However, results of previous studies were still inconsistent and inconclusive. For example, a meta-analysis indicated that wearable activity trackers were associated with a significant reduction of 35.46 min/d sedentary time among hospitalized patients, but this finding was only from 2 studies included in this review. Other two systematic reviews found that smartphone-based interventions had opposite results in interrupting SB in older adults, whereas this evidence was only obtained from limited studies (n ≤2) in these reviews; and the definition of SB was different.  Given the rapid development in digital technologies and variation of intervention patterns, as well as the importance of improving SB among chronic diseases patients, it is crucial to update and comprehensively summarize the evidence to incorporate digital solutions for this vulnerable population”. |
| Objectives | 4 | Provide an explicit statement of the objective(s) or question(s) the review addresses. | Abstract part:  “Objective: This review aimed to evaluate the effectiveness of digital health interventions in reducing SB among patients with chronic diseases”.  In the end of the “Introduction” part:  “ Thereby, this systematic review and meta-analysis aimed to search and synthesize available evidence regarding the effectiveness of digital health interventions in improving SB among patients with chronic diseases”. |
| **METHODS** | | |  |
| Eligibility criteria | 5 | Specify the inclusion and exclusion criteria for the review and how studies were grouped for the syntheses. | Inclusion and Exclusion Criteria part:  “The PICOS framework was used to select eligible studies. Participants (P): adults diagnosed with chronic diseases in terms of cardiovascular disease, chronic respiratory disease, cancer, diabetes, metabolic syndrome (MetS), obesity, rheumatoid arthritis (RA), stroke, and other chronic conditions that defined by the WHO. Intervention (I): Digital health intervention is a discrete functionality of digital technology that is applied to achieve health objectives according to WHO guideline. Therefore, interventions utilized any type of digital technologies, consisting of mobile phones, web, software applications, wearable trackers, emails, or other digital technologies, were included. Studies that exclusively employed wearable trackers to measure SB were excluded. Meanwhile, the intervention components included approaches targeting SB directly, or PA, which may have a compensatory SB reduction. Control (C): any comparison groups without digital technologies, including usual care, wait-list, active control or blank control. Outcome (O): The definition of SB from Sedentary Behavior Research Network was adopted. Any SB related outcomes, including overall sitting time, pre-post sitting time changes, SB proportion, sedentary bouts and breaks of prolonged sitting, which was assessed at baseline and endpoint using objective or subjective measures, either as primary or secondary outcomes, were involved for analysis. Study type (S): randomized controlled trial (RCT). Those pilot RCTs, feasibility studies, protocol papers, brief report of RCTs, studies with sample size <10 and studies with unavailable full-text were excluded”. |
| Information sources | 6 | Specify all databases, registers, websites, organisations, reference lists and other sources searched or consulted to identify studies. Specify the date when each source was last searched or consulted. | Search method part:  “The search strategy was developed by one author (YZ) and then reviewed and finalized by two experts (YJX and FWN) (Supplementary material, S1). Two keywords were first defined to develop Medical Subject Headings (Mesh) terms in the search strategy: sedentary behavior and digital technology. In order to find out patients with any potential chronic illness, no key word in particular for any chronic disease was used in the search strategy. We systematically searched for and retrieved all articles that focused on SB interventions utilizing digital health technologies, and then screened out the studies targeting patients with chronic diseases. At last, a comprehensive search was carried out in PubMed, Embase, Scopus, Web of Science, CINAHL Complete, Cochrane Library, and ACM Digital Library. The articles in English published from 2000 onwards were included, which aligned with the first release of the WHO’s document on the approach to digital health strategies”. |
| Search strategy | 7 | Present the full search strategies for all databases, registers and websites, including any filters and limits used. | “The search strategy could be found in Supplementary material, S1”. See details in Supplementary material, S1. |
| Selection process | 8 | Specify the methods used to decide whether a study met the inclusion criteria of the review, including how many reviewers screened each record and each report retrieved, whether they worked independently, and if applicable, details of automation tools used in the process. | Study Selection and data extraction part:  “Duplicate studies were identified and removed using the Note-express software. The remaining studies were screened independently by two authors (YZ and QLY) in a sequential manner of title, abstract, and full-text screening. Conflicts were settled by consulting a third author (YJX)”. |
| Data collection process | 9 | Specify the methods used to collect data from reports, including how many reviewers collected data from each report, whether they worked independently, any processes for obtaining or confirming data from study investigators, and if applicable, details of automation tools used in the process. | Study Selection and data extraction part:  “A standardized data extraction form was used to collect the data of study characteristics, intervention details, and outcomes”. |
| Data items | 10a | List and define all outcomes for which data were sought. Specify whether all results that were compatible with each outcome domain in each study were sought (e.g. for all measures, time points, analyses), and if not, the methods used to decide which results to collect. | Study Selection and data extraction part:  “A standardized data extraction form was used to collect data about the basic characteristics, intervention details, and outcomes”. See details in Table 1. |
|  | 10b | List and define all other variables for which data were sought (e.g. participant and intervention characteristics, funding sources). Describe any assumptions made about any missing or unclear information. | Study Selection and data extraction part:  “Study characteristics encompassed the first author, publication year, country, category of chronic diseases, and participant characteristics (such as group size, age, sex distribution, and dropout rates). Intervention details included the components of the interventions, duration, and type of digital technology. The outcome parameters, including SB, were obtained”. |
| Study risk of bias assessment | 11 | Specify the methods used to assess risk of bias in the included studies, including details of the tool(s) used, how many reviewers assessed each study and whether they worked independently, and if applicable, details of automation tools used in the process. | Study quality assessment part:  “The risk of bias was assessed using the ‘Revised Cochrane risk-of-bias tool for randomized trials’ (RoB2). Five domains with signaling questions were evaluated, including the randomization process, the effect of assignment and adherence to the intervention, missing outcome data, outcome measurement, and the selection of reported results. Each criterion was assessed and categorized as ‘low risk’, ‘some concern’, or ‘high risk’ of bias. An algorithm based on these five domains was utilized to determine the overall bias. Two independent reviewers (YZ and QLY) evaluated the selected articles, and any disagreements were resolved by a third party (YJX)”. |
| Effect measures | 12 | Specify for each outcome the effect measure(s) (e.g. risk ratio, mean difference) used in the synthesis or presentation of results. | Statistical analysis part:  “The mean difference (MD) with a random or fixed effect model was employed to compare the pooled effect across studies. Cohen’s d was used to assess the effect size, with d >0.8 indicating a large effect, 0.5-0.8 a medium effect, and 0.2-0.5 a small effect”. |
| Synthesis methods | 13a | Describe the processes used to decide which studies were eligible for each synthesis (e.g. tabulating the study intervention characteristics and comparing against the planned groups for each synthesis (item #5)). | “One study reported an average sedentary time of 18.8 hours based on objective measurement, which was clinically unreasonable and might include sleeping time. Therefore, the subjective data on sedentary time was extracted in this study”. |
|  | 13b | Describe any methods required to prepare the data for presentation or synthesis, such as handling of missing summary statistics, or data conversions. | “Generally, objective measurement data with higher accuracy was preferred for meta-analysis. However, in one article, the objective measurement indicated an average sedentary time of 18.8 hours, which was not clinically plausible and may include sleeping time. As a result, only subjective data on sedentary time was extracted and included in the meta-analysis”.  “Among these four studies, one of them presented unreasonable data regarding the breaks without explanation. Therefore, only three RCTs were included in the meta-analysis”. |
|  | 13c | Describe any methods used to tabulate or visually display results of individual studies and syntheses. | “ Forest plots were utilized to visually compare and display the estimated effects and confidence intervals. Funnel plots were employed to evaluate publication bias when there were at least 10 studies in the meta-analysis”. |
|  | 13d | Describe any methods used to synthesize results and provide a rationale for the choice(s). If meta-analysis was performed, describe the model(s), method(s) to identify the presence and extent of statistical heterogeneity, and software package(s) used. | Statistical analysis part:  “The data analysis was conducted using Review Manager 5.3”.  “And the heterogeneity in studies was assessed by I^2^, categorized as low (I^2^ <25%), medium (25%≤ I^2^ <75%), or high (I^2^ ≥75%)”. |
|  | 13e | Describe any methods used to explore possible causes of heterogeneity among study results (e.g. subgroup analysis, meta-regression). | “Subgroup analyses were conducted based on disease classification, age groups, theoretical basis, primary/compensatory SB reduction, intervention duration, and objective/subjective measurement of SB”. |
|  | 13f | Describe any sensitivity analyses conducted to assess robustness of the synthesized results. | “Sensitivity analyses with leave-one-out were conducted to assess the robustness of the main results”. |
| Reporting bias assessment | 14 | Describe any methods used to assess risk of bias due to missing results in a synthesis (arising from reporting biases). | “Funnel plots were employed to evaluate publication bias when there were at least 10 studies in the meta-analysis”. |
| Certainty assessment | 15 | Describe any methods used to assess certainty (or confidence) in the body of evidence for an outcome. | “Cohen’s d was used to assess the effect size, with d >0.8 indicating a large effect, 0.5-0.8 a medium effect, and 0.2-0.5 a small effect”. |
| **RESULTS** | | |  |
| Study selection | 16a | Describe the results of the search and selection process, from the number of records identified in the search to the number of studies included in the review, ideally using a flow diagram. | “A total of 7842 studies were identified after the systematic search. After removing duplicates, 6337 articles were screened by title and abstract. At last, 338 were retrieved by full text for eligibility and 312 articles were excluded due to non-target population, design, and inconsistent definition of SB, like TV watching, physical inactivity, or steps (excluded studies details in Supplementary S1). Fig.1 shows the selection process. In total, 26 studies were identified”. |
|  | 16b | Cite studies that might appear to meet the inclusion criteria, but which were excluded, and explain why they were excluded. | “Among the included studies, four studies were based on two separately registered RCTs with different sample sizes or follow-up duration33-36, all the studies were then included. While for other three RCTs based on the same study37-39, only one was selected as representative, because it was closely matched the registered content, also published earlier, and directly focused on SB interventions. Details of the selected articles were summarized in Table 1)”. |
| Study characteristics | 17 | Cite each included study and present its characteristics. | “All the included studies were published in English between 2011 and 2023, with the majority in Western countries (16/26, 61.5%). The Netherlands and Australia had the same and highest number of studies (n=4). There were 2 three-arm RCTs and 1 four-arm RCT, all others were two-arm RCTs. The sample size varied from 51 to 622, in total 3800 participants were involved, with an average age of 57.32 (SD=9.91) years. There were more women (n=2042) than men (n=1758) in the total sample, while two studies specifically focused on women. The involved chronic diseases were obesity (n=6), RA (n=5), CAD (n=4), cancer (n=4), T2DM (n=3), MetS (n=2), and stroke (n=2)”. |
| Risk of bias in studies | 18 | Present assessments of risk of bias for each included study. | Risk of Bias part:  “The 80.8% (21/26) of the studies had a low to moderate risk of bias (Supplementary material, S2). Domains assessed as high risk for bias included missing outcome data and the selection of reported results. Three articles had no description of the allocation concealment. One study had unbalanced baseline data regarding marital status and employment status (P<0.05). One study reported longer sitting time in intervention group than the control group (9.8 vs 8.8 h/d) at baseline. No study adopted blinding because it was not possible due to the nature of the intervention. Three studies applied the on-protocol analysis, while the rest of the studies used the intention-to-treat analysis. For the domain of missing outcome data, only 8 of the studies reported a data availability ≥95%. Most of the studies were in line with existing published protocols, except for three articles. Two of them decreased sample size owing to the impact of COVID-19, and one modified the recruitment strategy as there were not enough individuals from the target population”. |
| Results of individual studies | 19 | For all outcomes, present, for each study: (a) summary statistics for each group (where appropriate) and (b) an effect estimate and its precision (e.g. confidence/credible interval), ideally using structured tables or plots. | *Yes, see details in the results part. Here is an example.*  *Effects of digital health interventions on reducing overall sitting time*  “Sixteen RCTs reported the overall sitting time (min/d) of each group, including 914 participants in the intervention group and 856 in the control group. The overall sitting time was significantly decreased with a pooled mean difference of 30.8 min/d (95% CI: -49.79, -11.82; I2=65%; P=0.001) after the interventions (Fig.2), with a small effect size (Cohen’s d=-0.27; 95% CI: -0.44, -0.11; Z=3.25; P=0.001). The study weight, mean difference of overall sitting time, and mean age of the participants were showed in a grouped bubble plot (Supplementary material, S3)”. |
| Results of syntheses | 20a | For each synthesis, briefly summarise the characteristics and risk of bias among contributing studies. | Yes. See details in the results part. |
|  | 20b | Present results of all statistical syntheses conducted. If meta-analysis was done, present for each the summary estimate and its precision (e.g. confidence/credible interval) and measures of statistical heterogeneity. If comparing groups, describe the direction of the effect. | “Four RCTs reported SB proportion results. The forest plot showed a pooled mean difference of 4.36% reduction in favor of the digital health interventions (95% CI: -6.39, -2.32; I2=20%; P<0.001) (Fig.4), with a small effect size (Cohen’s d=-0.39; 95% CI: -0.57, -0.20; Z=4.07; P<0.001)”. |
|  | 20c | Present results of all investigations of possible causes of heterogeneity among study results. | “Six studies reported the compensatory SB reduction by increasing PA level (without addressing SB directly), which included LPA/MVPA, CR, and free choice of any kinds of exercise, such as gardening, cycling, endurance and strength training. The heterogeneity was high (I2=84%).” |
|  | 20d | Present results of all sensitivity analyses conducted to assess the robustness of the synthesized results. | “Leave-one-out sensitivity analysis showed consistent associations between digital health interventions and overall sitting time, pre-post sitting time changes, SB proportion, sedentary bouts, and breaks of sedentary time, implying the robustness of the key results”. |
| Reporting biases | 21 | Present assessments of risk of bias due to missing results (arising from reporting biases) for each synthesis assessed. | Publication bias and sensitivity analysis part:  “There was no serious publication bias indicated by the symmetry observed in the funnel plot for overall sitting time (Supplementary material, S4)”. |
| Certainty of evidence | 22 | Present assessments of certainty (or confidence) in the body of evidence for each outcome assessed. | N/A |
| **DISCUSSION** | | |  |
| Discussion | 23a | Provide a general interpretation of the results in the context of other evidence. | “This systematic review and meta-analysis indicated that digital health interventions were effective in reducing sitting time (overall sitting time, pre-post sitting time changes and SB proportion) among patients with chronic diseases. The overall sitting time decreased by 30.8 min/d, which was less than the SB reduction (42.28min/d) observed in a previous meta-analysis, evaluating digital technology-enhanced interventions on SB among healthy adults in a workplace setting. This difference may be explained by different populations (healthy vs. unhealthy populations) and settings, which was supported by Gardner et al that workplace SB was more receptive to routinization than non-workplace SB”. |
|  | 23b | Discuss any limitations of the evidence included in the review. | “There were also certain limitations. Firstly, this review only selected articles published in English, which may exist a language bias. Secondly, the limited number of RCTs included in subgroup analyses posed challenge in identifying potential sources of bias. Thirdly, both the substantial heterogeneity observed among the interventions’ components and united use of digital technologies limited interpretation of the independent contribution of each approach or technology. While it is not feasible to conduct a meta-analysis to compare different technologies, the findings emphasized the potential effectiveness of different combinations of digital technologies in reducing SB. In addition, a few publications were identified as study protocols or pilot studies, necessitating their exclusion with the intent to update at a later time”. |
|  | 23c | Discuss any limitations of the review processes used. | “Caution should be exercised when interpreting the results of this review and meta-analysis, especially for the subgroup analyses with the limited number of articles”. |
|  | 23d | Discuss implications of the results for practice, policy, and future research. | “Recommendations for future study and clinical practice  In clinical practice, nurses could leverage phone, web or activity tracker-based interventions to offer support to sedentary patients with chronic diseases, which may effectively reduce their sitting time and then facilitate better disease management.  The availability of digital health interventions with rigorous design targeting SB reduction remains limited, particularly for certain type of chronic illnesses. Additionally, it is recommended to place a greater emphasis on addressing sedentary bouts and breaks, rather than solely focusing on reducing overall sitting time. Meanwhile, this review reveals a reduction in compensatory SB reduction following PA interventions, which has not been widely studied before. Further research is needed to understand the underlying mechanisms and its implications for behavior modification.” |
| **OTHER INFORMATION** | | |  |
| Registration and protocol | 24a | Provide registration information for the review, including register name and registration number, or state that the review was not registered. | This systematic review and meta-analysis was registered in the PROSPERO (No.CRD42023477958). |
|  | 24b | Indicate where the review protocol can be accessed, or state that a protocol was not prepared. | “This protocol could be found in the PROSPERO (No.CRD42023477958)” |
|  | 24c | Describe and explain any amendments to information provided at registration or in the protocol. | Initially, our protocol included the consideration of pilot RCTs in addition to full-scale RCTs. However, upon conducting the formal literature search, we discovered a substantial number of RCTs that met our inclusion criteria. As a result, we made the decision not to include pilot RCTs in our final analysis. The rationale behind this exclusion was based on the sufficiency of available full-scale RCTs, which adequately addressed our research question. By focusing solely on full-scale RCTs, we aimed to ensure a more comprehensive and robust evaluation of the intervention's effectiveness. |
| Support | 25 | Describe sources of financial or non-financial support for the review, and the role of the funders or sponsors in the review. | No funding. |
| Competing interests | 26 | Declare any competing interests of review authors. | No competing interests of all authors. |
| Availability of data, code and other materials | 27 | Report which of the following are publicly available and where they can be found: template data collection forms; data extracted from included studies; data used for all analyses; analytic code; any other materials used in the review. | “The search strategy could be found in Supplementary material, S1.” |

*From:*  Page MJ, McKenzie JE, Bossuyt PM, Boutron I, Hoffmann TC, Mulrow CD, et al. The PRISMA 2020 statement: an updated guideline for reporting systematic reviews. BMJ 2021;372:n71. doi: 10.1136/bmj.n71

For more information, visit: <http://www.prisma-statement.org/>
